# Cholinergic Activation of Corticofugal Circuits in the Adult Mouse Prefrontal Cortex

**DOI:** 10.1523/JNEUROSCI.1388-23.2023

**Published:** 2024-01-17

**Authors:** Allan T. Gulledge

**Affiliations:** Department of Molecular and Systems Biology, Geisel School of Medicine at Dartmouth College, Hanover 03755, New Hampshire

**Keywords:** acetylcholine, layer 5, muscarinic receptor, prefrontal cortex, pyramidal neuron, synaptic transmission

## Abstract

Acetylcholine (ACh) promotes neocortical output to the thalamus and brainstem by preferentially enhancing the postsynaptic excitability of layer 5 pyramidal tract (PT) neurons relative to neighboring intratelencephalic (IT) neurons. Less is known about how ACh regulates the excitatory synaptic drive of IT and PT neurons. To address this question, spontaneous excitatory postsynaptic potentials (sEPSPs) were recorded in dual recordings of IT and PT neurons in slices of prelimbic cortex from adult female and male mice. ACh (20 µM) enhanced sEPSP amplitudes, frequencies, rise-times, and half-widths preferentially in PT neurons. These effects were blocked by the muscarinic receptor antagonist atropine (1 µM). When challenged with pirenzepine (1 µM), an antagonist selective for M1-type muscarinic receptors, ACh instead reduced sEPSP frequencies, suggesting that ACh may generally suppress synaptic transmission in the cortex via non-M1 receptors. Cholinergic enhancement of sEPSPs in PT neurons was not sensitive to antagonism of GABA receptors with gabazine (10 µM) and CGP52432 (2.5 µM) but was blocked by tetrodotoxin (1 µM), suggesting that ACh enhances action-potential-dependent excitatory synaptic transmission in PT neurons. ACh also preferentially promoted the occurrence of synchronous sEPSPs in dual recordings of PT neurons relative to IT–PT and IT–IT parings. Finally, selective chemogenetic silencing of hM4Di-expressing PT, but not commissural IT, neurons blocked cholinergic enhancement of sEPSP amplitudes and frequencies in PT neurons. These data suggest that, in addition to selectively enhancing the postsynaptic excitability of PT neurons, M1 receptor activation promotes corticofugal output by amplifying recurrent excitation within networks of PT neurons.

## Significance Statement

ACh is a neurotransmitter that preferentially enhances the excitability of neocortical projection neurons targeting the brainstem (pyramidal tract, or PT neurons). The present study is significant in revealing that ACh also increases excitatory synaptic drive preferentially in PT neurons. Pharmacological and chemogenetic experiments demonstrate that ACh acts via M1-type muscarinic AChRs to activate networks of recurrently connected PT neurons. Thus, ACh may bias cortical output to the brainstem and other subcortical structures via parallel increases in excitatory synaptic drive and postsynaptic excitability in PT neurons.

## Introduction

Output from the neocortex is segregated into two broad, nonoverlapping channels based on the axonal projection patterns of pyramidal neuron subpopulations: intratelencephalic (IT) neurons provide the bulk of corticocortical projections, including interhemispheric projections, whereas pyramidal tract (PT) neurons provide corticofugal output to deep subcortical structures such as the thalamus and brainstem (for review, see Baker et al., 2018a). These pyramidal neuron subpopulations exist side-by-side in layer 5 of the neocortex, including the prelimbic cortex, an area of medial prefrontal cortex (mPFC) critical for decision making and goal-directed behavior (for review, see Sharpe et al., 2019; Green and Bouton, 2021).

A growing literature has revealed that IT and PT neurons are differentially sensitive to neuromodulators allowing for brain-state-dependent regulation of cortical circuit output (Dembrow et al., 2010; Avesar and Gulledge, 2012; Gee et al., 2012; Elliott et al., 2018). Experiments in this laboratory and others have found that ACh preferentially enhances the intrinsic excitability of PT neurons, relative to IT neurons, in juvenile (Joshi et al., 2016) and adult (Dembrow et al., 2010; Baker et al., 2018b) rodents via activation of postsynaptic M1-type muscarinic AChRs (mAChRs; Gulledge et al., 2009). Yet, rather than directly exciting PT neurons, ACh increases postsynaptic gain to amplify action potential output in response to excitatory synaptic drive (Andrade, 1991; Gulledge and Stuart, 2005; Williams and Fletcher, 2019). Therefore, understanding the net effect of ACh on cortical circuit output will require determination of how ACh regulates excitatory synaptic transmission in IT and PT neurons.

Prior studies have found that ACh acts presynaptically to suppress glutamate release at many synapses in the neocortex (Vidal and Changeux, 1993; Gil et al., 1997; Tsodyks and Markram, 1997; Hsieh et al., 2000; Atzori et al., 2005; Levy et al., 2006) and hippocampus (Hounsgaard, 1978; Valentino and Dingledine, 1981; Segal, 1982; Hasselmo and Schnell, 1994; Hasselmo et al., 1995; Fernandez de Sevilla et al., 2002; Goswamee and McQuiston, 2019). The inhibitory effect of ACh on excitatory synaptic transmission is attributable primarily to activation of M4-type mAChRs (Kimura and Baughman 1997; Shirey et al., 2008; Eggermann and Feldmeyer, 2009; Dasari and Gulledge, 2011; Yang et al., 2020). On the other hand, ACh may enhance glutamate release at some cortical synapses via presynaptic nicotinic AChRs (nAChRs; Gil et al., 1997; Aramakis and Metherate, 1998; Urban-Ciecko et al., 2018; Yang et al., 2020). ACh may also increase (Yang et al., 2020) or decrease (Atzori et al., 2005) the frequency of “miniature” glutamatergic synaptic events in neocortical neurons, and can increase the frequency and amplitude of action-potential-dependent spontaneous excitatory events in layer 5 neurons of the mPFC (Shirey et al., 2009).

Aside from the substantial evidence for muscarinic suppression of evoked transmitter release at cortical synapses, results in the studies described above are variable and inconsistent. This may reflect developmental differences in cholinergic signaling (Aramakis and Metherate, 1998), as most of the studies described above utilized neurons spanning from young to adolescent animals (8 d old to ∼4 weeks old). Alternatively, variation may arise from differences in cortical areas studied, species differences (e.g., mice vs rats; Elliott et al., 2018), or differences in other experimental parameters. Of note, only one prior study tested for projection-specific cholinergic modulation of excitatory drive (Yang et al., 2020), doing so in two populations of layer 6 projection neurons from immature (≤3 weeks old) animals.

The present study was designed to compare the impact of ACh on the net excitatory drive of layer 5 IT and PT neurons in the adult mPFC. Given the considerable evidence for muscarinic suppression of glutamate release across diverse experimental conditions, it was hypothesized that ACh should reduce both the frequency and amplitudes of spontaneous excitatory postsynaptic potentials (sEPSPs) in IT and PT neurons. As detailed below, this hypothesis was tested using simultaneous recordings of sEPSPs in pairs of IT and PT neurons in slices of the adult mouse prelimbic cortex.

## Methods

### Animals and ethical approvals

Experiments were performed using female and male 6- to 16-week-old (mean ± SD of 10.4 ± 2.5-week-old, *n* = 51) C57BL/6J wild-type mice cared for under protocols approved by the Institutional Animal Care and Use Committee of Dartmouth College. Animals were bred and maintained on a 12 h light–dark cycle with free access to food and water in facilities accredited by the Association for Assessment and Accreditation of Laboratory Animal Care.

### Animal surgeries

For some experiments, an AAV retrograde virus expressing pAAV-hSyn-hM4D(Gi)-mCherry (Addgene #50475) or pAAV-hSyn-hM3D(Gq)-mCherry (Addgene #50474) was injected into either the ipsilateral pons (relative to lambda, 1.00 mm lateral, 0.20 mm posterior, depth of 4.55 mm) or contralateral mPFC (relative to bregma, 0.48 mm lateral, 2.1 mm anterior, depth of 1.6 mm) of 6- to 9-week-old mice to express DREADD (“designer receptor exclusively activated by designer drugs”) receptors selectively in IT (contralateral mPFC injections) or PT (ipsilateral pons injections) neurons in the prefrontal cortex. Under sterile surgical conditions, animals were anesthetized with continuous isoflurane (∼2%) and a craniotomy made at the above coordinates. A 33-gauge microsyringe (Hamilton) containing undiluted virus was slowly lowered into place over a period of ∼5 min. After waiting anther 5 min, the virus was injected at a rate of 50 nl per min for a total volume of 450 nl (pons) or 300 nl (mPFC). Five min after the injection was completed, the microsyringe was slowly removed and the wound sutured. Mice were allowed to recover from surgery for at least 21 d before use in experiments.

### Slice preparation

Mice were anesthetized with vaporized isoflurane and decapitated. Brains were rapidly removed and sliced in an artificial cerebral spinal fluid (aCSF) composed of (in mM): 125 NaCl, 25 NaHCO_3_, 3 KCl, 1.25 NaH_2_PO_4_, 0.5 CaCl_2_, 6 MgCl_2_, 0.01 ascorbic acid, and 25 glucose (saturated with 95% O_2_/5% CO_2_). Coronal brain slices (250 µm thickness) containing the mPFC were stored in a holding chamber filled with normal recording aCSF containing 2 mM CaCl_2_ and 1 mM MgCl_2_ for 1 h at 35°C and then maintained at room temperature (∼26°C) until use in experiments.

### Electrophysiology

Slices were transferred to a recording chamber (∼0.5 ml volume) perfused continuously (∼6 ml/min) with oxygenated aCSF heated to 35–36°C. Layer 5 pyramidal neurons in the prelimbic cortex were visualized using oblique illumination with a 60x water-immersion objective (Olympus). Whole-cell recordings were made of layer 5 neurons using patch pipettes (5–7 MΩ) filled with a solution containing (in mM): 140 K-gluconate, 2 NaCl, 2 MgCl_2_, 10 HEPES, 3 Na_2_ATP, and 0.3 NaGTP, pH 7.2 with KOH. Data were acquired in a current-clamp mode using a two-electrode amplifier (dPatch, Sutter Instrument) connected to a Mac Studio computer (Apple, Inc.) running SutterPatch software under Igor Pro (Sutter Instrument). The capacitance was maximally neutralized and series resistances (typically 15–25 MΩ) were compensated with bridge balance. Membrane potentials were sampled at 25 kHz (250 kHz for action potential waveforms), filtered at 5 kHz (50 kHz for action potentials), and corrected for the liquid junction potential (+12 mV). Experiments were conducted in current-clamp mode to allow for physiological classification of IT and PT neurons and to avoid the distortion of synaptic events intrinsic to voltage-clamp (Williams and Mitchell, 2008; Beaulieu-Laroche and Harnett, 2018).

**Figure 1.**
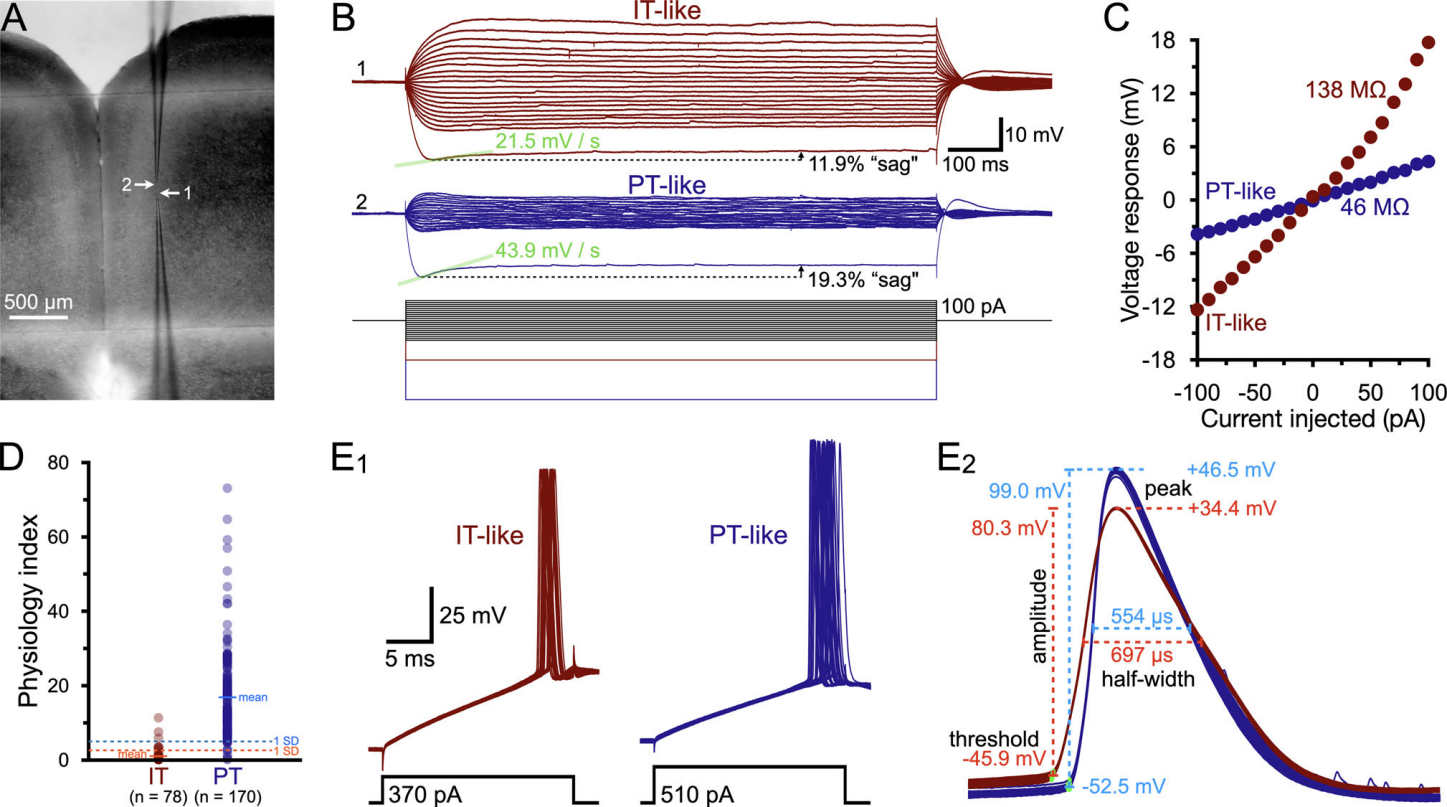
Physiological classification of intratelencephalic (IT) and pyramidal tract (PT) layer 5 projection neurons. ***A***, Image of a coronal slice of mouse brain containing the medial prefrontal cortex (5× objective) showing the locations of two recording electrodes in layer 5. ***B***, Voltage responses in the two neurons recorded in ***A*** (top and middle traces) to a series of current steps (lower traces). Note that neuron 1 (red, IT-like) has larger voltage deflections to the current steps, a correspondingly larger input resistance, a smaller depolarizing “sag” response after a 20 mV hyperpolarizing step (indicative of limited HCN channel activation), and a smaller slope of the initial sag response than observed in neuron 2 (blue, PT-like). These parameters were used to compute a PI (see Methods) of 1.85 and 18.6 for neurons 1 and 2, respectively. ***C***, Plots of the voltage-current relationships for data shown in ***B***. Linear regressions from −50 to +50 pA were used to calculate the input resistances of these neurons. ***D***, PIs calculated for 248 retrograde-labeled commissural projection neurons (a type of IT neuron) and corticopontine neurons (a type of PT neuron) from Baker et al. (2018b). Labeled IT neurons (*n* = 78) had a mean (±SD) PI of 0.85 ± 1.72, whereas labeled PT neurons (*n* = 170) had a mean PI of 16.9 ± 11.9. ***E_1_***, Brief (20 ms) current steps were used to evoke action potentials captured at 250 kHz (30 superimposed trials) in the IT-like and PT-like neurons shown in ***B***,***C***. ***E_2_***, Magnification of the peak-aligned action potentials shown in ***E_1_***, showing measurements made of spike thresholds, amplitudes, peaks, and widths at half-amplitude (“half-widths”), as listed in Table 2.

IT and PT neurons can be distinguish based on their physiological profiles that differ especially with regard to expression of hyperpolarization-activated and cyclic nucleotide-gated (HCN) channels, which are expressed at a higher level in PT neurons relative to IT neurons (Sheets et al., 2011; Avesar and Gulledge, 2012; Oswald et al., 2013). Layer 5 neurons in this study were classified as either IT or PT based on their physiological profiles (Table 1, Fig. 1) as quantified by a physiology index (PI) that successfully identified 93% of retrograde-labeled projection neurons from an earlier study (Baker et al., 2018b; see Fig. 1*D*). Similar physiological measures have been used by others to sort IT and PT neurons in the mPFC (Lee et al., 2014; Elliott et al., 2018; Meda et al., 2019). For each neuron, the PI was computed based on the magnitude of the HCN-mediated rebound “sag” potential (as a percent of the peak hyperpolarization following a current pulse sufficient to generate a peak hyperpolarization of ∼20 mV below the resting membrane potential), the “slope of sag” rebound (as measured by linear regression over a 50 ms period beginning at the time of peak hyperpolarization), and the input resistance (*R_N_*) of the neuron, according to the following formula:

$$\mathrm{PI}\;=\;\frac{\mathrm{sag}\;(\%)\times \mathrm{slope}\;\mathrm{of}\;\mathrm{sag}\left(\frac{\mathrm{mV}}{s}\right)}{R_{N}\;(M\Omega )}.$$

**Table 1.** Physiological classification of IT and PT neurons for each experiment

Neuron group	Neuron type	*n*	*R_N_* (MΩ)	Sag (%)	Sag slope (mV/s)	Physiology index
Figure 3	IT	19	165 ± 84	7.6 ± 3.6	13 ± 8	0.7 ± 0.6
	PT	19	56 ± 12	18.7 ± 4.5	50 ± 16	18.5 ± 11.7
Figure 4	PT	10	58 ± 15	19.2 ± 3.8	50 ± 15	18.6 ± 10.2
Figure 5	PT	13	49 ± 12	16.0 ± 3.0	38 ± 10	13.3 ± 5.8
Figure 6	PT	13	45 ± 11	16.9 ± 4.6	38 ± 10	15.9 ± 9.8
Figure 7	PT	16	67 ± 17	21.3 ± 6.2	49 ± 16	17.6 ± 12.1
Figure 8	IT	24	145 ± 57	9.2 ± 3.5	16 ± 9	1.2 ± 0.9
	PT	44	57 ± 15	17.6 ± 4.1	46 ± 16	15.6 ± 8.5
Figure 9	PT^a^	21	51 ± 15	18.1 ± 4.8	42 ± 15	17.0 ± 10.8
Figure 10	PT	12	53 ± 7	17.3 ± 3.3	36 ± 5	12.1 ± 3.8
Figure 11	PT^a^	9	43 ± 11	16.9 ± 3.9	34 ± 11	14.5 ± 6.4
All neurons	IT	43	154 ± 70	8.5 ± 3.6	15 ± 9	1.0 ± 0.8
	PT	157	54 ± 15	18.0 ± 4.5	44 ± 15	16.0 ± 9.4

Data are shown as means ± SD. Statistical treatments are not applicable, as IT and PT populations were defined based on measurements of *R_N_*, % sag, and sag slope (i.e., the Physiology Index; see Methods).

^a^ Targeted PT neurons retrograde-labeled with hM4Di- or hM3Dq-mCherry.

When calculated for retrograde-labeled neurons in a previous study (Baker et al., 2018b), layer 5 IT neurons projecting to the contralateral hemisphere (*n* = 78) had a mean (±SD) PI of 0.85 ± 1.72, while labeled pons-projecting PT neurons (*n* = 170) had a mean PI of 16.9 ± 11.9. In this study, neurons were classified as “IT” if their calculated PI was less than 2.6 (one SD above the mean for labeled IT neurons in Baker et al., 2018b) or “PT” if their PIs were greater than 5.0 (one SD below the mean of labeled PT neurons in the previous study). Neurons with PIs between 2.6 and 5.0 were considered ambiguous and excluded from analysis. When tested against labeled commissural (IT) and brainstem projecting (PT) neurons from another previous paper (Avesar and Gulledge, 2012), this classification scheme misidentified only 1 of 43 (2.3%) neurons. The classification scheme also correctly identified all 30 retrograde-labeled PT neurons studied in this present report (Table 1).

For each neuron, additional physiological measurements were made of the resting membrane potential (*V_M_*), the amplitude of afterhyperpolarizations (AHPs; see below), and action potential (AP) attributes, including AP threshold (as detected with a 50 mV/ms threshold), peak voltages, amplitudes from threshold, and widths at half-amplitudes (i.e., half-widths; Table 2, Fig. 1*E*). AHPs were generated with a train of brief (2 ms) high amplitude (3 nA) current pulses delivered at 50 Hz for 3 s (Gulledge et al., 2013), and the peak hyperpolarization during the AHP was measured relative to the initial resting *V_M_*. To measure AP kinetics, single APs were evoked with 20-ms current pulses (typically in the range of 300–500 pA) and sampled at 250 kHz.

**Table 2.** Additional physiological properties of IT and PT neurons in each experiment

Neuron group	Neuron type	*n*	Resting *V_M_* (mV)	AHP amplitude (mV)	AP threshold (mV)	AP peak (mV)	AP amplitude (mV)	AP half-width (ms)
Figure 3	IT	19	−83.6 ± 3.8	−6.3 ± 3.1	−49.3 ± 4.3	+44.5 ± 6.1	93.8 ± 7.5	604 ± 104
	PT	19	−79.5 ± 2.7	−7.1 ± 1.2	−54.1 ± 3.7	+45.7 ± 8.0	103.8 ± 6.7	558 ± 61
	IT vs PT *t* test^b^ (*p*)		<0.001	0.269	<0.001	0.088	0.001	0.068
	Effect size (*d*)		**1.26**	0.35	**1.19**	**0.79**	**1.41**	0.53
Figure 4	PT	10	−80.4 ± 2.9	−7.1 ± 0.7	−55.1 ± 3.0	+48.5 ± 3.9	103.6 ± 4.8	524 ± 32
Figure 5	PT	13	−80.1 ± 2.4	−6.6 ± 1.2	−53.2 ± 2.6	+46.5 ± 4.6	98.4 ± 6.4	507 ± 70
Figure 6	PT	13	−79.1 ± 2.5	−7.1 ± 1.1	−55.5 ± 2.2	+46.4 ± 2.6	101.8 ± 3.3	460 ± 53
Figure 7	PT	16	−77.7 ± 2.4	−7.1 ± 1.1	−52.4 ± 3.7	+45.9 ± 7.6	98.3 ± 8.6	565 ± 70
Figure 8	IT	24	−82.5 ± 3.2	−5.8 ± 1.4	−47.0 ± 4.9	+43.9 ± 6.4	90.9 ± 6.8	686 ± 112
	PT	44	−78.9 ± 3.2	−6.6 ± 1.6	−52.4 ± 3.2	+47.0 ± 6.3	99.3 ± 7.3	553 ± 79
	IT vs PT *t* test^c^ (*p*)		<0.001	0.033	<0.001	0.065	<0.001	<0.001
	Effect size (*d*)		**1.30**	0.60	**1.54**	0.55	**1.33**	**1.64**
Figure 9	PT^a^	21	−79.2 ± 2.3	−6.9 ± 0.7	−54.9 ± 2.0	+44.3 ± 4.2	99.1 ± 4.4	459 ± 59
Figure 10	PT	12	−81.4 ± 3.3	−7.7 ± 1.3	−55.8 ± 2.9	+43.2 ± 3.8	99.0 ± 4.8	478 ± 44
Figure 11	PT^a^	9	−76.9 ± 4.8	−7.5 ± 1.0	−55.2 ± 1.6	+44.7 ± 2.7	100.0 ± 2.8	435 ± 32
All neurons	IT	43	−83.0 ± 3.5	−6.0 ± 2.3	−48.0 ± 4.7	+44.1 ± 6.2	92.1 ± 7.2	652 ± 115
	PT	157	−79.9 ± 3.8	−7.0 ± 1.3	−53.8 ± 3.2	+46.3 ± 5.6	100.1 ± 6.3	515 ± 77
	IT vs PT *t* test^c^ (*p*)		<0.001	0.013	<0.001	0.046	<0.001	<0.001
	Effect size (*d*)		**0.87**	0.61	**1.65**	0.38	**1.22**	**1.58**

Data presented as means ± SD. Large effect sizes (≥∼0.80) are shown in bold.

^a^ Targeted PT neurons retrograde-labeled with hM4Di- or hM3Dq-mCherry.

^b^ Student's *t* tests for paired samples were used for comparisons of means of physiological attributes for dual recordings from IT and PT neurons.

^c^ Student's *t* tests assuming unequal variances were used for comparisons of physiological attributes of IT and PT neurons recorded as homotypic pairs and for the combined data sets.

Following baseline physiological measurements, spontaneous excitatory postsynaptic potentials (sEPSPs) were recorded over 30 min with neurons “held” at their resting *V_M_* using the dPatch “dynamic holding” function that introduces a bias current to counteract slow changes in *V_M_* due to ACh or other drugs (e.g., clozapine n-oxide; CNO) even as much faster synaptic potentials are faithfully recorded. While the use of bias current to hold postsynaptic neurons at their initial resting membrane potentials is ideal for measuring sEPSPs, it obscures slower intrinsic membrane potential dynamics that may be induced by cholinergic modulation. However, quantifying the ACh-induced bias current (i.e., the “holding current”) over time provides a useful alternative measure of the postsynaptic impact of ACh (or other substances) on IT and PT neurons.

**Figure 2.**
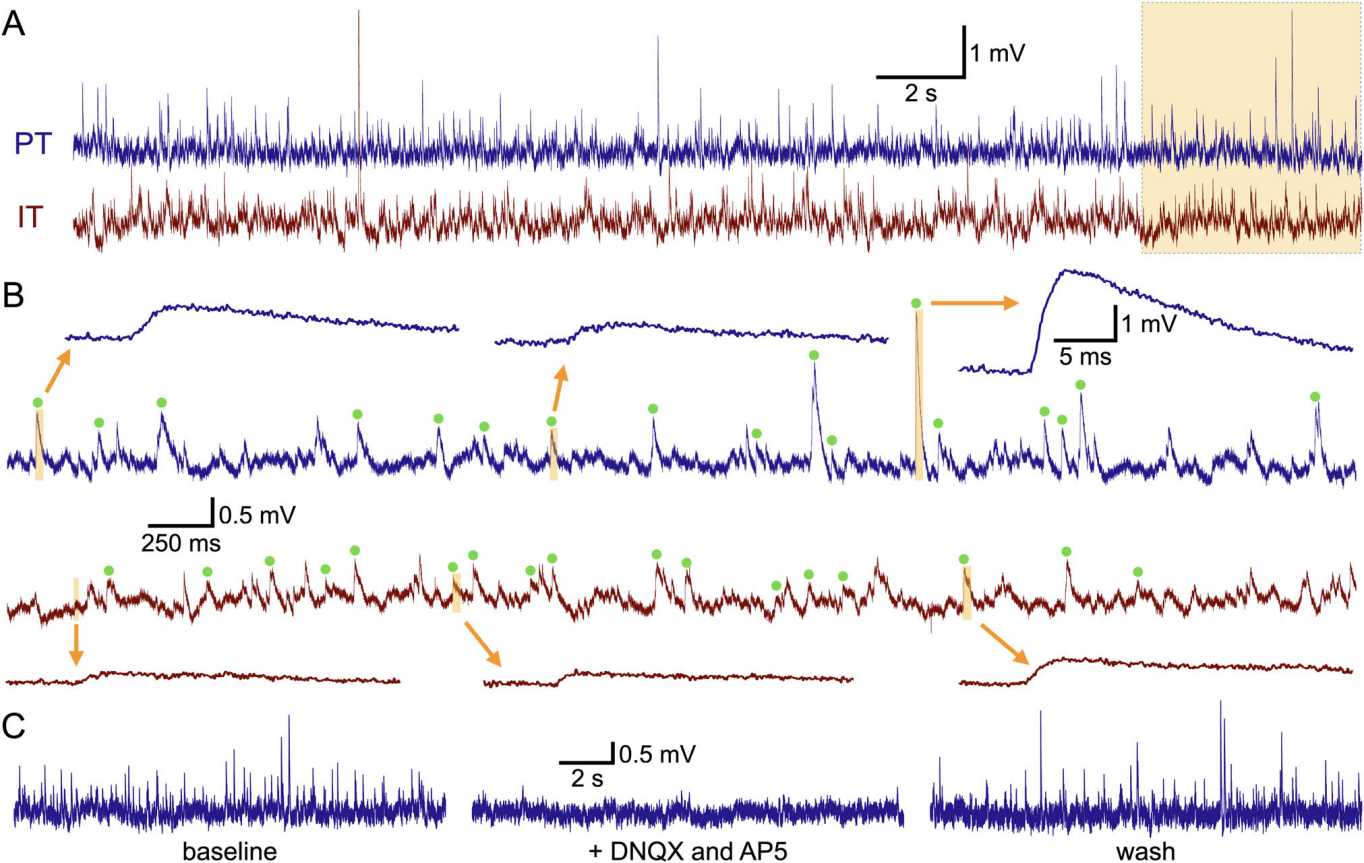
Detection of spontaneous excitatory postsynaptic potentials (sEPSPs) in IT and PT neurons. ***A***, Recordings of membrane potentials in a pair of IT and PT neurons. The final 5 s (orange shading) are expanded in ***B****.* ***B***, Green dots show sEPSPs detected by the sEPSP template (see Methods). Several captured sEPSPs are shown at an expanded timescale. ***C***, In a different PT neuron, 15-s-long traces of spontaneous activity in baseline conditions (left) and after co-application of the glutamate receptor blockers DNQX (10 µM) and D-AP5 (25 µM; middle). sEPSPs suppressed by glutamate receptor antagonism returned after washing off the drugs (right).

**Figure 3.**
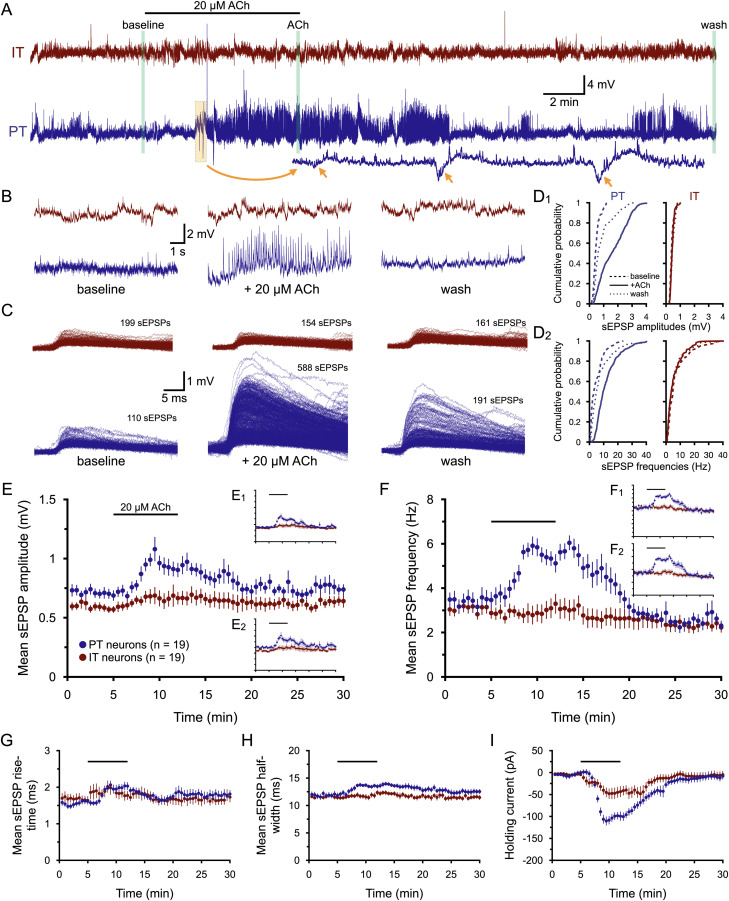
ACh selectively enhances excitatory synaptic input to PT neurons. ***A***, In a pair of IT (red) and PT (blue) neurons, membrane potentials were recorded at the soma for 30 min (shown at a resampled rate of 1 kHz). After 5 min of baseline recording, 20 µM ACh (with 10 µM eserine) was bath applied for 7 min. Inset at bottom (orange arrows) are enlarged spontaneous hyperpolarizations occurring in the PT neuron that greatly resemble M1-receptor-driven and SK-potassium-channel-mediated responses to ACh (Gulledge and Stuart, 2005). ***B***, Expanded 10 s traces of membrane potentials taken at the end of the baseline period (left), after 7 min of ACh exposure (middle), and at the end of the experiment (right), as indicated by green bars in ***A****.* ***C***, Superposition of all sEPSPs detected in the final 1-min periods of baseline recording (left), ACh exposure (middle), and wash (right) in the IT (red) and PT (blue) neurons. ***D***, Cumulative probability plots of sEPSP amplitudes (***D_1_***) and instantaneous sEPSP frequencies (***D_2_***) for the sEPSPs shown in ***C***. ***E***, Plots of mean sEPSP amplitudes (±SEM) over time for 19 pairs of simultaneously recorded IT (red) and PT (blue) neurons (30 s means). Insets show the same data with EPSPs detected at 2.5x standard deviation of noise (***E_1_***) or with an EPSP template with 50% slower kinetics (***E_2_***). ***F***, Similar to ***E***, including insets, but plotting mean sEPSP frequencies over time. Note that the *y*-axis in ***F_1_*** extends to 12 Hz. ***G*–*I***, Plots of mean sEPSP rise-times (***G***), widths at half-amplitude (***H***), and the holding currents necessary to keep neurons at their resting membrane potentials (***I***) over time.

During recordings, ACh and/or other substances were bath-applied for periods of time (typically 7 min for bath-applied ACh). Data were analyzed offline using AxoGraph or Igor Pro. Each 30-min sweep of spontaneous activity was high-pass filtered at 0.2 Hz, low-pass filtered at 5 kHz and notch-filtered at 60 Hz. Unless otherwise specified, sEPSPs were detected and captured using an EPSP template with a 2 ms rise and an 18 ms decay with the threshold set to five times the noise standard deviation (SD) to minimize any chance of false-positive events (see Fig. 2*A,B*). sEPSP peaks were measured as the average amplitude across a 0.3 ms period centered on the peak of the sEPSP. sEPSP frequencies were determined for each 30-s period by dividing the number of detected events by 30. Event detection was set to find EPSPs with at least a 10-ms inter-event interval, and to accept the first of two or more overlapping EPSPs, although occasionally two EPSPs occurring very close in time will be considered a single event. For consistency, and to preserve non-biased analysis, detection and measurement of all EPSPs was automated using the same parameters, unless otherwise indicated (e.g., insets in Fig. 3*E*), and comparisons of sEPSP attributes and holding currents were made from mean values in the final 1 min of baseline conditions (or during the final 1 min of exposure to cholinergic antagonists, tetrodotoxin [TTX], or CNO) and during the final 1 min of exposure to ACh (or CNO), regardless of where the peak effects of ACh or CNO were observed.

sEPSPs in dual recordings were considered “synchronous” if their onsets occurred within a 0.5 ms window. The incidence of synchronous sEPSPs as a proportion (%) of all sEPSPs was determined for each 30 s time period, and results for each neuron in a dual recording were averaged to generate a pair-associated incidence of synchronous sEPSPs (i.e., the number of dual recordings = “*n*”; Fig. 8*C*, red, purple, and blue bars). Because some sEPSPs in dual recordings may occur by chance within a 0.5 ms window, the incidence of synchronous events in dual recordings was compared with the incidence of chance synchronous sEPSPs detected in simulated pairings of sEPSP timings obtained from neurons from other recording sessions. To do this, EPSP timings for each neuron of a dual recording were compared with the timings of sEPSPs occurring in all other neurons in the same experimental group (IT–PT, IT–IT, or PT–PT; Fig. 8*C*, green bars). For instance, for each of the 22 PT–PT dual recordings in Figure 8 (44 PT neurons in total), the incidence of “by chance” synchronous sEPSPs were determined for all 84 possible simulated pairings (i.e., each neuron in a given PT–PT dual recording had 42 simulated partners from other dual PT–PT recordings) and the incidence of synchronous sEPSPs from these 84 simulated pairings, as a proportion of all sEPSPs, were averaged together for each 30 s period to determine the mean “chance” level of synchronous events for that original dual recording (Fig. 8*C*, green bars). This process was repeated for each dual-recording pair, allowing for pair-wise comparisons of the rate of actual synchronous events in each 30 s period (*n* = 22) with the corresponding mean rate of chance synchronous events for that pair (*n* = 22). Simulated pairings of sEPSP timings were also generated for IT–IT (*n* = 12 dual recordings) and IT–PT (*n* = 19 dual recordings) data sets, with simulated pairings from IT–PT dual recordings limited to heterotypic (IT–PT) pairs. This allowed determination of whether the incidence of synchronous sEPSPs in actual dual recordings rose above chance levels in each 30 s bin. To determine the impact of experimental condition (baseline, ACh, and wash) on the occurrence of synchronous sEPSPs, data were averaged over the final 4 min of each condition (eight 30 s bins).

### Drugs

Most drugs were dissolved in water to make stock solutions (typically at 10,000× concentration) that were frozen as aliquots and used as needed during experiments. ACh (Fisher Scientific) was bath-applied with physostigmine hemisulfate (eserine; Tocris Bioscience), a blocker of acetylcholinesterase (AChE). Atropine and pirenzepine dihydrochloride were purchased from Sigma-Aldrich. TTX citrate and CGP 52432 were purchased from HelloBio, Inc. CNO dihydrochloride, DNQX, and D-AP5 were purchased from Tocris Bioscience. SR-95531 (gabazine) was purchased from MedChemExpress and dissolved in DMSO for stock solutions.

### Experimental design and statistical analysis

When data are descriptive and convey variability within populations (e.g., physiological properties of IT and PT neurons), data are presented as means ± SDs. When data are used to compare mean values (e.g., in plots of sEPSP attributes) data are presented as means ± standard error of the mean (SEM). Within group mean effects of drug treatments (e.g., ACh) and comparisons of effects in pairs of neurons (e.g., in IT–PT dual recordings) used two-tailed Student's *t* tests for paired samples. Comparisons of effects across unpaired groups utilized two-tailed Student's *t* tests for samples with unequal variance. Because of the limited usefulness of *p*-values from statistical tests (Nieuwenhuis et al., 2011; Wasserstein et al., 2019), no assumptions are made of *p*-value “significance” (i.e., *α*). Instead, to better quantify the meaningfulness of physiological differences observed in IT and PT neurons, and the impact of ACh on sEPSP attributes, effect sizes are reported as Cohen's *d*, a measure of the standardized mean difference between groups or conditions, according to the following formula:

$$d\;=\;\frac{\left|\left({\bar{x}}_{A}-{\bar{x}}_{B}\right)\right|}{\sqrt{\frac{\left((n_{A}-1){\mathrm{SD}}_{A}^{2}\;+\;(n_{B}-1){\mathrm{SD}}_{B}^{2}\right)}{(n_{A}\;+\;n_{B}-2)}}}\;$$

where *x̅_A_* and *x̅_B_* are the mean values of a parameter (e.g., sEPSP amplitude), *SD_A_* and *SD_B_* are the standard deviations for those parameters, and *n_A_* and *n_B_* are the number of neurons (or neuron pairs) in each group (e.g., IT neurons vs PT neurons) or condition (e.g., baseline conditions and in the presence of ACh). By convention, effect sizes ∼0.8 or larger are considered as having “large” impact (i.e., with an overlap of distributions of only ∼50% or less), and values of ∼0.5 and ∼0.2 considered as having “medium” or “small” impacts, respectively, according to their larger (~67% and ~85%, respectively) overlaps in distributions (Cohen, 1988).

## Results

### ACh enhances spontaneous EPSPs preferentially in PT neurons

As previously described in this laboratory and others (Dembrow et al., 2010; Joshi et al., 2016; Baker et al., 2018b), ACh preferentially enhances the intrinsic excitability of neocortical PT neurons relative to neighboring IT neurons to promote corticofugal output to the thalamus and brainstem. What is less established is how ACh regulates the afferent excitatory drive of these two projection neuron subtypes. To quantify the net impact of ACh on overall excitatory drive, simultaneous dual whole-cell recordings were made in pairs of layer 5 pyramidal neurons in the mouse prelimbic cortex (mean distance [±SD] between somata was 56 ± 25 µm [*n* = 53 dual recordings described below]). IT and PT neurons were classified based on their physiological responses to depolarizing and hyperpolarizing current steps (see Methods, Fig. 1*A–D*, and Table 1). Identified IT and PT neurons also differed in additional physiological measures, including their resting *V_M_*, the peak amplitudes of afterhyperpolarizations, and action potential (AP) attributes, including spike thresholds, peaks, amplitudes, and half-widths (Fig. 1*E* and Table 2). For both IT and PT populations, physiological attributes were similar in neurons from female (*n* = 78 neurons) and male (*n* = 128 neurons) mice.

Based on previous work showing that ACh suppresses glutamate release at some corticocortical synapses in the neocortex (Vidal and Changeux, 1993; Gil et al., 1997; Tsodyks and Markram, 1997; Hsieh et al., 2000; Atzori et al., 2005; Levy et al., 2006), it was hypothesized that exposure to ACh would reduce the amplitude and frequency of sEPSPs in IT and PT neurons in the prelimbic cortex. To test this, sEPSPs were monitored continuously in 19 pairs of IT (mean PI of 0.74 ± 0.64 [SD]) and PT (mean PI of 18.5 ± 11.7) neurons in layer 5 of the mouse prelimbic cortex over a 30-min period during which ACh (20 µM, with 10 µM eserine, a blocker of AChE) was bath applied for 7 min (Fig. 3*A*). The glutamatergic nature of these sEPSPs was confirmed in four experiments in which bath application of 10 µM DNQX (an AMPA receptor blocker) and 25 µM D-AP5 (an NMDA receptor blocker) reversibly reduced the number of detected sEPSPs by 97 ± 1% (Fig. 2*C*).

Contrary to expectations, ACh *increased* sEPSP amplitudes and frequencies preferentially in PT neurons (Fig. 3*A–F*; Table 3). Despite their lower input resistances, mean sEPSP amplitudes tended to be larger in PT neurons relative to IT neurons regardless of experimental condition (Table 3), whereas other sEPSP attributes, including mean frequencies, rise-times, and half-widths, were similar in the two neuron subtypes in baseline conditions (Table 3). Population time-courses of mean sEPSP attributes and holding currents are shown in Fig. 3*E–I* for 19 IT and PT neuron dual recordings. The effects of ACh peaked about 5 min into the 7 min exposure and typically plateaued or slightly decreased in magnitude during the final 2 min in ACh before returning to near-baseline values by the end of the 30 min recording. For example, peak increases in sEPSP amplitudes and frequencies occurred, on average, after 4.9 ± 0.6 min and 5.1 ± 0.5 min, respectively, of ACh exposure in IT neurons, and at 4.3 ± 0.9 min and 5.4 ± 0.5 min, respectively, in PT neurons. ACh also occasionally triggered spontaneous hyperpolarizations (inset in Fig. 3*A*; 9 of 19 IT neurons, 9 of 19 PT neurons) that resemble cholinergic responses mediated by SK-type potassium channels in response to M1 receptor stimulation, as previously characterized in detail in neocortical (Gulledge and Stuart, 2005; Gulledge et al., 2007, 2009; Dasari et al., 2017; Baker et al., 2018b) and hippocampal (Gulledge and Kawaguchi, 2007; Dasari and Gulledge, 2011) pyramidal neurons. As such, these responses were not further characterized in the current study.

**Table 3.** Effects of ACh on sEPSPs in IT–PT pairs (*n* = 19)

Measurement	Neuron type	Baseline	+ACh	Wash	Change in ACh	ACh vs baseline^a^ (*p*-value)	Effect size (*d*)
sEPSP amplitude (mV)	IT	0.57 ± 0.03	0.68 ± 0.08	0.64 ± 0.05	+16 ± 6%	0.056	0.44
	PT	0.69 ± 0.04	0.91 ± 0.06	0.74 ± 0.04	+38 ± 13%	0.007	**0.94**
	IT–PT *t* test^a^ (*p*)	0.013	0.020	0.063	0.157		
	Effect size (*d*)	**0.79**	**0.80**	0.48	0.50		
sEPSP frequency (Hz)	IT	3.0 ± 0.3	3.2 ± 0.4	2.3 ± 0.3	+9 ± 10%	0.386	0.19
	PT	3.5 ± 0.4	5.2 ± 0.3	2.5 ± 0.2	+92 ± 24%	<0.001	**1.26**
	IT–PT *t* test^a^ (*p*)	0.200	<0.001	0.648	0.014		
	Effect size (*d*)	0.33	**1.24**	0.14	**0.93**		
sEPSP rise-time (ms)	IT	1.65 ± 0.11	1.77 ± 0.13	1.75 ± 0.15	+10 ± 6%	0.249	0.24
	PT	1.62 ± 0.08	2.03 ± 0.11	1.79 ± 0.11	+29 ± 7%	<0.001	**1.02**
	IT–PT *t* test^a^ (*p*)	0.	0.109	0.864	0.039		
	Effect size (*d*)	0.07	0.52	0.05	0.68		
sEPSP half-width (ms)	IT	11.5 ± 0.4	12.1 ± 0.5	11.5 ± 0.4	+6 ± 3%	0.051	0.33
	PT	11.9 ± 0.5	13.5 ± 0.5	12.5 ± 0.5	+16 ± 5%	0.003	**0.80**
	IT–PT *t* test^a^ (*p*)	0.394	0.027	0.140	0.056		
	Effect size (*d*)	0.24	**0.79**	0.54	0.62		
Holding current (pA)	IT	−4 ± 3	−44 ± 12	−6 ± 8	−40 ± 12 pA	0.004	**1.02**
	PT	−1 ± 5	−99 ± 9	−10 ± 7	−98 ± 8 pA	<0.001	**3.03**
	IT–PT *t* test^a^ (*p*)	0.605	<0.001	0.355	<0.001		
	Effect size (*d*)	0.18	**1.15**	0.13	**1.28**		

Data are shown as means ± SEM. Large effect sizes (≥∼0.80) are shown in bold.

^a^ Student's *t* test for paired samples.

For the 19 pairs of IT and PT neurons tested, ACh reversibly increased sEPSP amplitudes by 16 ± 6% (mean ± SEM) and 38 ± 13% in IT and PT neurons, respectively, and reversibly increased sEPSP frequencies selectively in PT neurons by 92 ± 24% (Table 3) compared with 9 ± 10% in IT neurons. The preferential impact of ACh on sEPSPs in PT neurons was not dependent on sEPSP detection threshold (Fig. 3*E_1_*,*F_1_*) or the kinetics of the EPSP template used to detect sEPSPs (Fig. 3*E_2_*,*F_2_*). ACh also preferentially increased sEPSP rise-times and half-widths in PT neurons, by 29 ± 7% and 16 ± 5%, respectively, compared to much smaller changes in IT neurons (mean changes of +10 ± 6 and +6 ± 3 for rise-time and half-width, respectively, in IT neurons; Fig. 3*G,H* and Table 3). Finally, ACh generated greater depolarizing currents in PT neurons, as reflected in the larger holding currents (−98 ± 8 pA vs −40 ± 12 pA for IT neurons) necessary to keep PT neurons at their initial resting *V_M_* during ACh exposure (Fig. 3*I* and Table 3). This preferential postsynaptic action of ACh on PT neuron excitability is consistent with previous reports finding that ACh preferentially excites PT neurons (Dembrow et al., 2010; Joshi et al., 2016; Baker et al., 2018b). The data presented here go further in demonstrating that ACh also preferentially increases the excitatory synaptic drive of PT neurons, suggesting that ACh will promote corticofugal output from the cortex via parallel enhancement of synaptic excitation and increased postsynaptic gain.

### Cholinergic enhancement of sEPSPs requires M1-type muscarinic receptors

**Figure 4.**
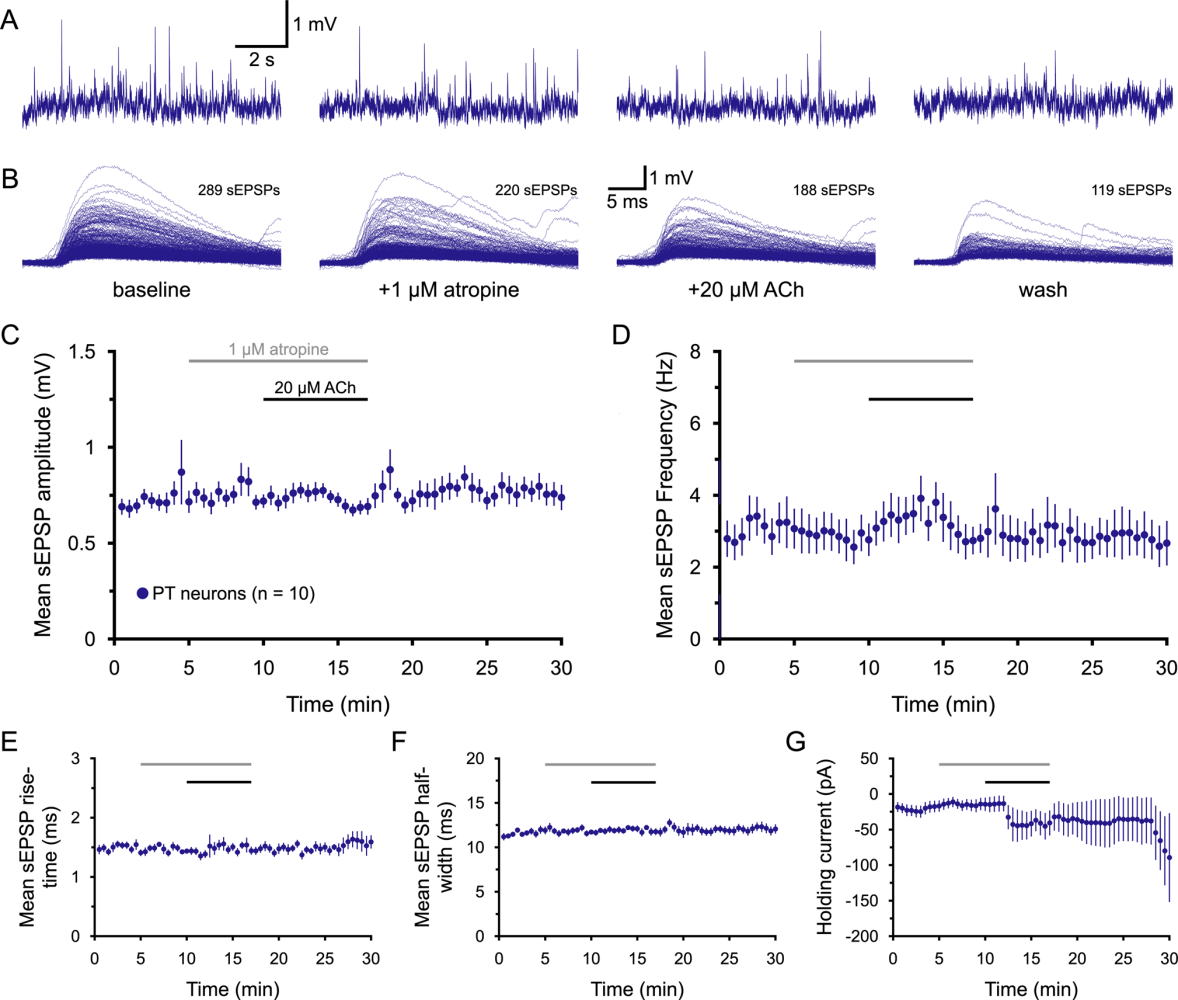
Cholinergic enhancement of sEPSPs in PT neurons is mediated by mAChRs. ***A***, Ten-second-long samples of membrane potential from a PT neuron taken at the end of the baseline recording period (far left), after 5 min of exposure to 1 µM atropine (a muscarinic receptor antagonist; middle left), after an additional 7 min of exposure to atropine and 20 µM ACh (with 10 µM eserine; middle right), and after 13 min of wash (far right), as indicated in ***B***. ***B***, Superimposed sEPSPs detected in the final 1-min periods of baseline recording, atropine-only exposure, atropine + ACh exposure, and wash, as indicated, for the neuron shown in ***A***. ***C***,***D***, Plots of mean sEPSP amplitude (***C***) and frequency (***D***) over time for 10 PT neurons exposed to atropine and ACh (30 s means). ***E*–*G***, Plots of mean sEPSP rise-time (***E***), width at half-amplitude (***F***), and the holding current necessary to keep neurons at their resting membrane potentials (***G***) over time.

In the mPFC, most postsynaptic effects of ACh in layer 5 pyramidal neurons are mediated by mAChRs (Gulledge et al., 2009; Hedrick and Waters, 2015). To test whether mAChRs are involved in regulating the excitatory drive of PT neurons, atropine (1 µM), a potent mAChR antagonist was bath applied for 5 min prior to additional application of ACh (20 µM, with eserine; Fig. 4). Atropine on its own had little impact on sEPSP attributes or holding currents (Table 4), but its presence blocked cholinergic modulation of sEPSPs and limited the amount of postsynaptic holding current necessary to keep neurons at their initial resting *V_M_* (*n* = 10 PT neurons; Fig. 4, Table 4). These data demonstrate that cholinergic enhancement of sEPSPs in PT neurons is mediated by mAChRs.

**Table 4.** Effects of ACh on sEPSPs in the presence of atropine (*n* = 10 PT neurons)

Measurement	Baseline	+Atropine	+ACh	Wash	Change in ACh	ACh vs atropine^a^ (*p*-value)	Effect size (*d*)
sEPSP amplitude (mV)	0.79 ± 0.12	0.72 ± 0.04	0.69 ± 0.04	0.75 ± 0.06	−3 ± 4%	0.257	0.24
sEPSP frequency (Hz)	3.2 ± 0.6	2.9 ± 0.5	2.7 ± 0.4	2.6 ± 0.6	+1 ± 10%	0.543	0.13
sEPSP rise-time (ms)	1.48 ± 0.08	1.43 ± 0.03	1.50 ± 0.07	1.56 ± 0.14	+5 ± 6%	0.424	0.39
sEPSP half-width (ms)	11.9 ± 0.4	11.6 ± 0.2	11.8 ± 0.3	12.0 ± 0.5	+2 ± 3%	0.602	0.19
Holding current (pA)	−17 ± 8	−14 ± 9	−43 ± 18	−85 ± 54	−29 ± 17 pA	0.119	0.64

Data are shown as means ± SEM.

^a^ Student's *t* test for paired samples.

**Figure 5.**
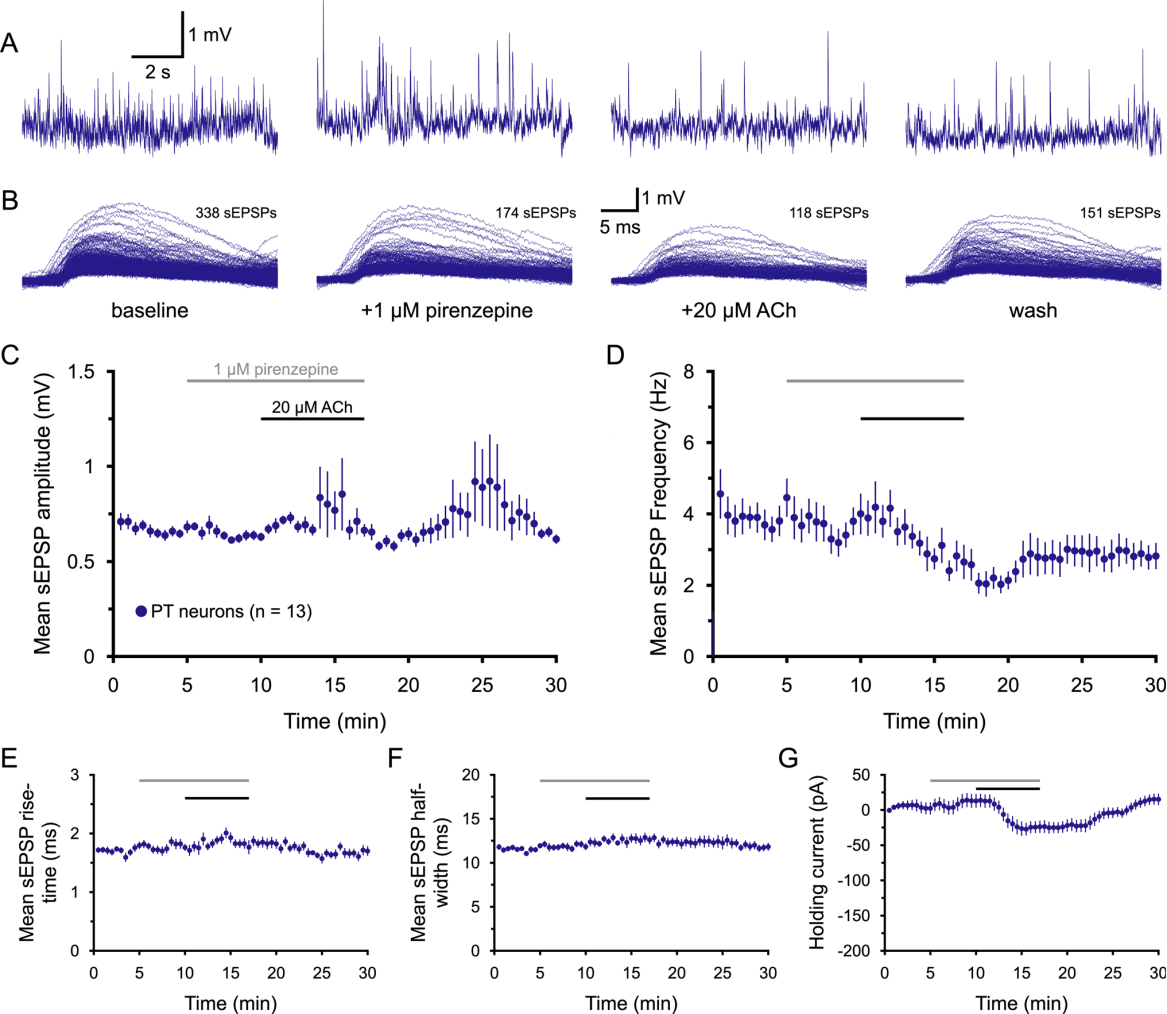
Cholinergic enhancement of sEPSPs in PT neurons is mediated by M1-type mAChRs. ***A***, Ten-second-long samples of membrane potential from a PT neuron taken at the end of the baseline recording period (far left), after 5 min of exposure to 1 µM pirenzepine (an antagonist for M1 muscarinic receptors; middle left), after an additional 7 min of exposure to pirenzepine and 20 µM ACh (with 10 µM eserine; middle right), and after 13 min of wash (far right), as indicated in ***B***. Superimposed sEPSPs detected in the final 1-min periods of baseline recording, pirenzepine-only exposure, pirenzepine + ACh exposure, and wash for the neuron shown in ***A***. ***C***,***D***, Plots of mean sEPSP amplitude (***C***) and frequency (***D***) over time for 13 PT neurons exposed to pirenzepine and ACh (30 s means). Note that, in the presence of pirenzepine, ACh *reduced*, rather than enhanced, sEPSP frequency in PT neurons. ***E***–***G***, Plots of mean sEPSP rise-time (***E***), width at half-amplitude (***F***), and the holding current necessary to keep neurons at their resting membrane potentials (***G***) over time.

It was previously reported that activation of M1-type mAChRs can increase the frequency and amplitude of excitatory postsynaptic currents (EPSCs) in layer 5 pyramidal neurons in the rodent prefrontal cortex (Shirey et al., 2009). To determine whether the cholinergic enhancement of sEPSPs in PT neurons depends on M1 receptors, ACh was applied in the presence of the M1-selective antagonist pirenzepine (PZP; 1 µM). As was the case for atropine, PZP on its own had little impact on sEPSP attributes or holding currents (*n* = 13 PT neurons; Fig. 5, Table 5). However, PZP blocked the ability of ACh to enhance sEPSP attributes (Fig. 5, Table 5) and reduced ACh-induced holding currents relative to the 19 control PT neurons from Figure 3 (*p* < 0.001, *d* = 2.1 for comparison of holding currents vs 19 control PT neurons). Instead, ACh in the presence of PZP *reduced* the frequency of sEPSPs by 20 ± 18% (from 3.9 ± 0.4 Hz to 2.7 ± 0.4 Hz; Table 5). This mean reduction was influenced by one neuron that exhibited a burst of activity in the last minute of ACh application. In the absence of that one neuron, the mean reduction in sEPSP frequency for the remaining 12 neurons was 37 ± 6%. The reversal of the cholinergic effect on sEPSP frequencies by PZP is similar to that reported by Shirey et al. (2009) and suggests that ACh may act broadly to suppress glutamate release from cortical afferents via M2 and/or M4 receptors (Kimura and Baughman 1997; Shirey et al., 2008; Eggermann and Feldmeyer, 2009; Dasari and Gulledge, 2011; Yang et al., 2020), but that this effect is normally masked in PT neurons by stronger M1-mediated enhancement of sEPSPs.

**Table 5.** Effects of ACh on sEPSPs in the presence of pirenzepine (PZP; *n* = 13 PT neurons)

Measurement	Baseline	+PZP	+ACh	Wash	Change in ACh	ACh vs PZP^a^ (*p*-value)	Effect size (*d*)
sEPSP amplitude (mV)	0.66 ± 0.02	0.63 ± 0.02	0.69 ± 0.05	0.64 ± 0.02	+10 ± 9%	0.328	0.41
sEPSP frequency (Hz)	4.1 ± 0.4	3.9 ± 0.4	2.7 ± 0.4	2.8 ± 0.3	−20 ± 18%	0.032	**0.83**
sEPSP rise-time (ms)	1.78 ± 0.06	1.79 ± 0.07	1.80 ± 0.10	1.71 ± 0.08	0 ± 5%	0.959	0.01
sEPSP half-width (ms)	11.7 ± 0.2	12.0 ± 0.3	12.7 ± 0.4	11.8 ± 0.4	+7 ± 3%	0.074	0.57
Holding current (pA)	+2 ± 9	−13 ± 10	−24 ± 9	+15 ± 8	−37 ± 7 pA	<0.001	**1.11**

Data are shown as means ± SEM. Large effect sizes (≥∼0.80) are shown in bold.

^a^ Student's *t* test for paired samples.

### Enhancement of sEPSPs by ACh does not require changes in GABAergic circuits

**Figure 6.**
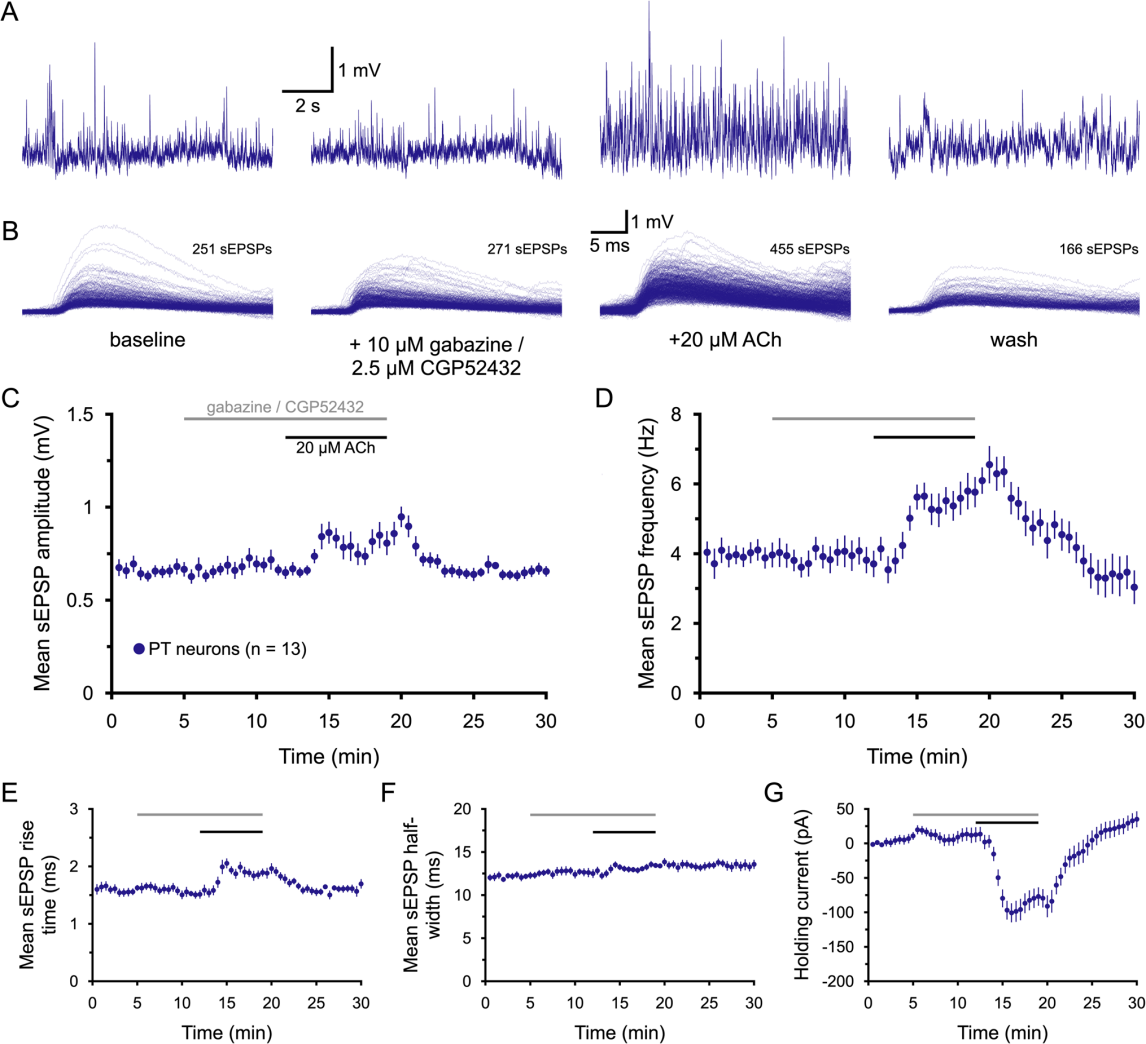
ACh enhancement of excitatory drive does not require modulation of GABAergic circuits. ***A***, Ten-second-long traces of membrane potential from a PT neuron taken at the end of the baseline recording period (far left), after 7 min of exposure to 10 µM gabazine and 2.5 µM CGP52432 (antagonists of GABA_A_ and GABA_B_ receptors, respectively; middle left), after an additional 7 min of exposure to GABA receptor blockers and 20 µM ACh (with 10 µM eserine; middle right), and after 11 min of wash (far right), as indicated in ***B***. Superimposed sEPSPs detected in the final 1-min periods of baseline recording, after GABA blockade, the addition of ACh, and after wash for the neuron shown in ***A***. ***C***,***D***, Plots of mean sEPSP amplitude (***C***) and frequency (***D***) over time for 13 PT neurons exposed to GABA blockers and ACh (30 s means). ***E***–***G***, Plots of mean sEPSP rise-time (***E***), width at half-amplitude (***F***), and the holding current necessary to keep neurons at their resting membrane potentials (***G***) over time.

In the recording conditions employed, GABA-mediated synaptic responses are expected to reverse very close to the resting *V_M_* (Gulledge and Stuart, 2003) and therefore likely do not contribute to measured sEPSPs (see also Fig. 2*C*). However, ACh might regulate GABAergic circuits in the cortex that influence the net excitatory drive of PT neurons (e.g., cholinergic disinhibition of excitatory circuits; Kruglikov and Rudy, 2008). To test whether ACh affects sEPSPs in PT neurons via modulation of GABAergic inhibition, blockers of both GABA_A_ (gabazine, 10 µM) and GABA_B_ (CGP52432 [CGP], 2.5 µM) receptors were bath applied for 7 min prior to additional application of ACh (*n* = 13; Fig. 6). Gabazine and CGP had little if any effect on sEPSPs or holding currents on their own, and their presence did not block cholinergic enhancement of sEPSP attributes or postsynaptic holding currents in PT neurons (Fig. 6, Table 6). In the presence of GABA receptor blockers, ACh enhanced sEPSP amplitudes and frequencies in PT neurons by 28 ± 10% and 67 ± 20%, respectively, amounts similar to those observed in PT neurons from IT–PT pairs in control conditions (compare with Table 3; *p* = 0.547, *d* = 0.20 for mean changes in sEPSP amplitude when compared to data from the 19 PT neurons in Figure 3; *p* = 0.485, *d* = 0.23 for changes in frequency). Thus, cholinergic enhancement of excitatory drive to PT neurons does not require modulation of GABAergic synaptic transmission.

**Table 6.** Effects of ACh on sEPSPs in the absence of GABAergic transmission (*n* = 13 PT neurons)

Measurement	Baseline	+Gabazine/CGP52432	+ACh	Wash	Change in ACh	ACh vs gabazine^a^ (*p*-value)	Effect size (*d*)
sEPSP amplitude (mV)	0.67 ± 0.04	0.66 ± 0.03	0.83 ± 0.07	0.66 ± 0.03	+28 ± 10%	0.018	**0.93**
sEPSP frequency (Hz)	3.9 ± 0.3	3.8 ± 0.4	5.8 ± 0.5	3.3 ± 0.5	+67 ± 20%	0.002	**1.29**
sEPSP rise-time (ms)	1.59 ± 0.05	1.51 ± 0.07	1.87 ± 0.07	1.63 ± 0.09	+22 ± 5%	<0.001	**1.47**
sEPSP half-width (ms)	12.2 ± 0.4	12.4 ± 0.5	13.4 ± 0.4	13.4 ± 0.6	+7 ± 3%	0.019	0.60
Holding current (pA)	9 ± 6	12 ± 11	−78 ± 14	+33 ± 11	−90.5 pA	<0.001	**2.01**

Data are shown as means ± SEM. Large effect sizes (≥∼0.80) are shown in bold.

^a^ Student's *t* test for paired samples.

### ACh promotes action-potential-dependent excitatory transmission in PT neurons

**Figure 7.**
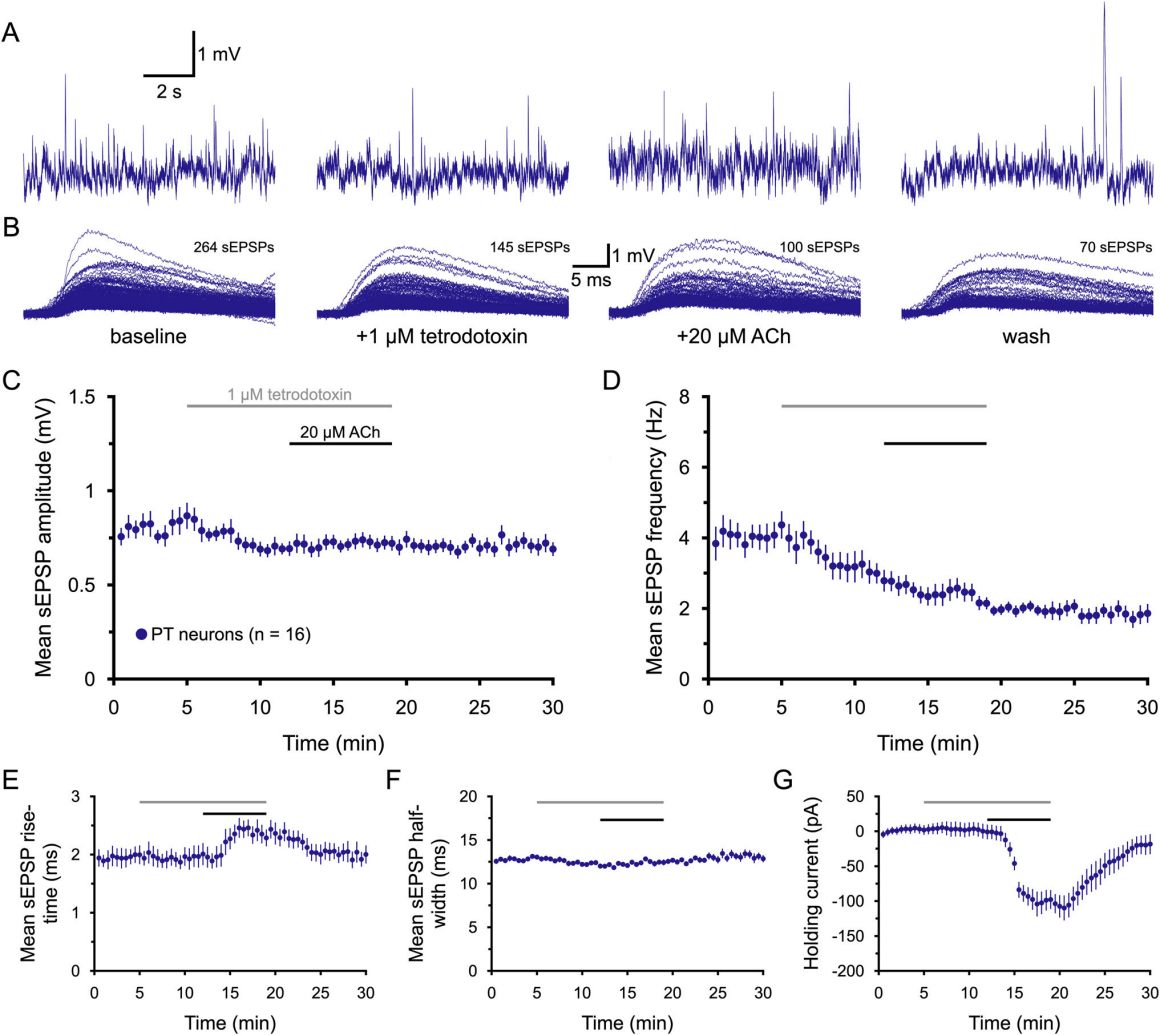
Cholinergic enhancement of excitatory synaptic drive requires action potentials. ***A***, Ten-second-long samples of membrane potential from a PT neuron taken at the end of the baseline recording period, after 7 min of exposure to 1 µM tetrodotoxin (TTX; a blocker of voltage-gated sodium channels), after an additional 7 min of exposure to TTX and 20 µM ACh (with 10 µM eserine), and after 11 min of wash, as indicated in ***B***. Superimposed sEPSPs detected in the final 1-min periods of baseline recording, TTX application, the additional application of ACh, and after 11 min of wash for the neuron shown in ***A***. ***C***,***D***, Plots of mean sEPSP amplitude (***C***) and frequency (***D***) over time for 16 PT neurons exposed to TTX and ACh (30 s bins). ***E***–***G***, Plots of mean sEPSP rise-time (***E***), width at half-amplitude (***F***), and the holding current necessary to keep neurons at their resting membrane potentials (***G***) over time. Note that, in the presence of TTX, the postsynaptic effect of ACh, as quantified by changes in holding current, remains robust. The ACh-induced increase in mEPSP rise-time in the absence of other changes suggests that this effect is also mediated by postsynaptic effects of ACh on electrotonic structure.

The “spontaneous” EPSPs described above comprise two distinct types of synaptic release. Some sEPSPs result from synaptic release following action potentials occurring spontaneously (i.e., not experimentally evoked) in the local network, while other sEPSPs reflect action-potential-independent “miniature” events occurring stochastically at individual synaptic boutons. To test whether ACh regulates one type of synaptic release or the other, TTX (1 µM), a blocker of voltage-gated sodium channels, was bath applied for 7 min prior to the additional application of ACh (*n* = 13; Fig. 7). Miniature EPSPs (mEPSPs) recorded in the presence of TTX were smaller (by an average of 14.9 ± 4.2%; *n* = 16 PT neurons; *p* = 0.028, *d* = 0.76), less frequent (by 30 ± 4%; *p* < 0.001, *d* = 0.96), and had shorter half-widths (by 6.3 ± 1.6%; *p* = 0.002, *d* = 0.96) than sEPSPs recorded in baseline conditions. On the other hand, TTX had little, if any, effect on sEPSP rise times (*p* = 0.904, *d* = 0.01) or holding currents (*p* = 0.639, *d* = 0.08). This suggests that 30% of sEPSPs recorded in baseline conditions reflect action-potential-dependent EPSPs that can be estimated to be, on average, ∼78% larger in amplitude (at ∼1.23 mV) than TTX-resistant mEPSPs (mean amplitude of 692 ± 32 µV; *n* = 16), leading to mean baseline sEPSP amplitudes averaging 854 ± 68 µV in the absence of TTX (*n* = 16; Table 7).

**Table 7.** Effects of ACh on miniature EPSPs (*n* = 16 PT neurons)

Measurement	Baseline	+TTX	+ACh	Wash	Change in ACh	ACh vs TTX^a^ (*p*-value)	Effect size (*d*)
sEPSP amplitude (mV)	0.85 ± 0.07	0.69 ± 0.03	0.72 ± 0.04	0.71 ± 0.04	+5 ± 5%	0.332	0.22
sEPSP frequency (Hz)	4.2 ± 0.4	2.9 ± 0.3	2.1 ± 0.2	1.8 ± 0.2	−19 ± 9%	0.005	0.69
sEPSP rise-time (ms)	2.00 ± 0.15	1.99 ± 0.19	2.32 ± 0.15	1.96 ± 0.14	+22 ± 6%	0.016	0.49
sEPSP half-width (ms)	13.1 ± 0.3	12.2 ± 0.2	12.4 ± 0.3	13.0 ± 0.5	+2 ± 2%	0.260	0.27
Holding current (pA)	+3 ± 7	0 ± 10	−98 ± 14	−19 ± 14	−98 ± 10 pA	<0.001	**1.96**

Data are shown as means ± SEM. Large effect sizes (≥∼0.80) are shown in bold.

^a^ Student's *t* test for paired samples.

More strikingly, the presence of TTX blocked cholinergic enhancement of synaptic transmission in PT neurons (Fig. 7, Table 7). Instead, mEPSP frequencies were *further reduced* by ACh, by an average of 19 ± 9% (Fig. 7*D*, Table 7), consistent with the idea that ACh acts broadly to suppress glutamate release at excitatory synapses via M2 and/or M4 receptors (see above). ACh in the presence of TTX continued to engage potent postsynaptic depolarizing currents (mean of −98 ± 10 pA) and increased mEPSP rise-times by 22 ± 6% (Fig. 7*E*,*G*, Table 7). These data demonstrate that ACh selectively enhances action-potential-dependent excitatory synaptic transmission in PT neurons, and that ACh-induced changes in sEPSP rise-times likely reflect postsynaptic changes in the electrotonic structure, rather than changes in synaptic release.

### ACh promotes synchronous input to PT neurons

**Figure 8.**
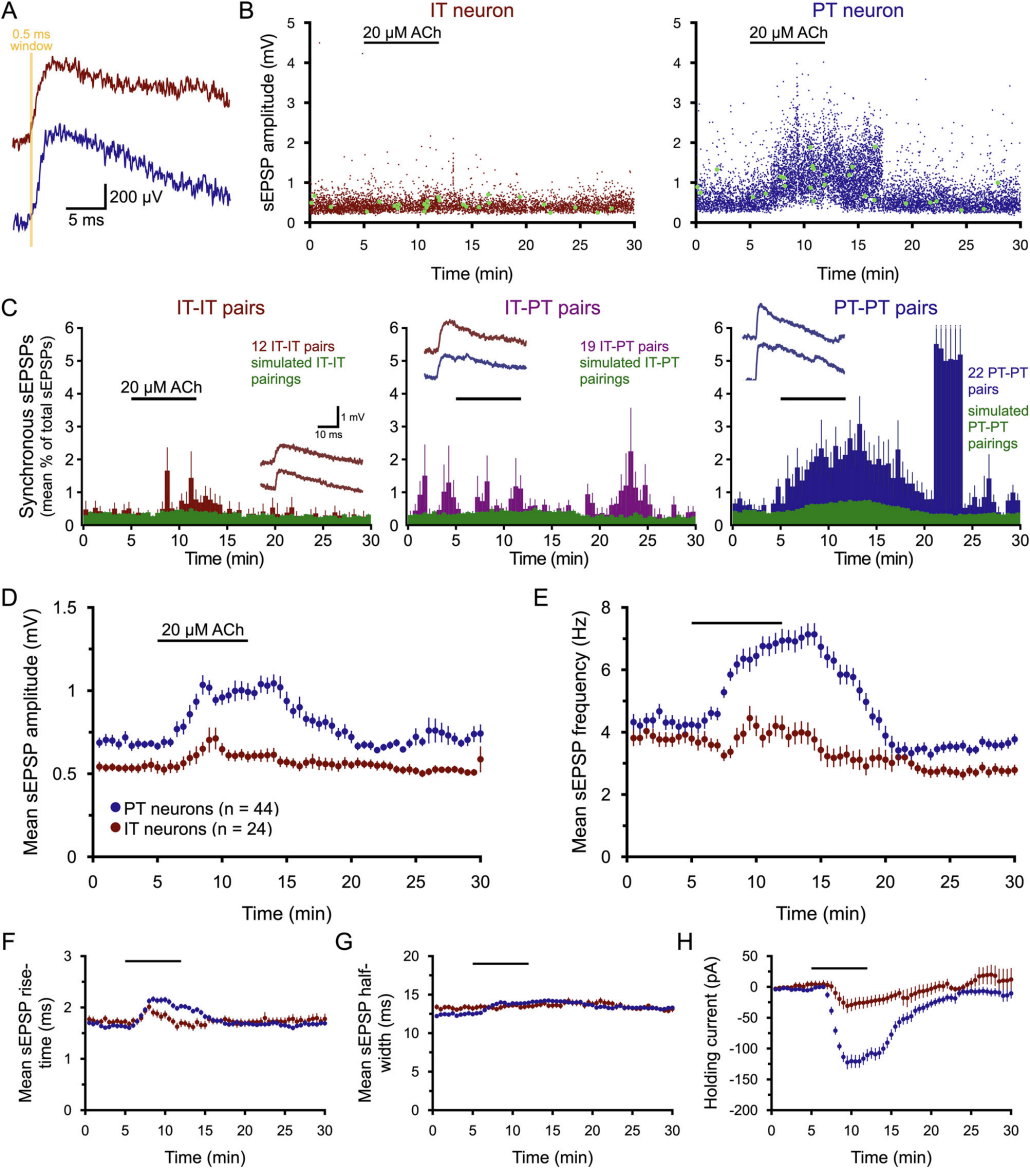
ACh promotes synchronous excitatory input in PT–PT neuron pairs. ***A***, A pair of “synchronous” sEPSPs initiating within an 0.5 ms window (yellow) detected in a pair of IT (red trace) and PT (blue trace) neurons. ***B***, Plots of sEPSP amplitudes over 30 min in simultaneous recordings of an IT (left) and PT (right) neuron. Green dots indicate synchronous EPSPs detected in both neurons. ***C***, Histograms showing the proportion (mean percentage ± SEM in 30 s bins) of all sEPSPs that occurred synchronously (i.e., with onsets within a 0.5 ms window) over time for pairs of IT neurons (left; red; *n* = 12 pairs), IT–PT pairs (middle; purple; the same 19 pairs as shown in Fig. 3), and PT–PT pairs (right; blue; *n* = 22 pairs). Green data indicate the proportions of sEPSPs (mean percentage ± SEM) detected as “synchronous” for all possible simulated pairings to provide a measure of “chance” likelihood of synchronous events over time (see Methods). Error bars for the burst of synchronous activity in PT–PT pairs (due to a large increase in one pair near minute 22) are truncated. Insets are examples of synchronous events from each pair-type. ***D***, Plots of mean sEPSP amplitudes (±SEM; 30 s means) over time for the 24 IT (red) and 44 PT (blue) neurons recorded in the homotypic pairs that contributed to panel ***C***. ***E***, Mean sEPSP frequencies over time for IT and PT neurons from homotypic pairs. ***F***–***H***, Plots of mean sEPSP rise-times (***F***), widths at half-amplitude (***G***), and the holding currents necessary to keep neurons at their resting membrane potentials (***H***) over time.

Unlike “miniature” synaptic transmission, which occurs stochastically at individual boutons, action-potential-dependent transmission is expected to promote transmitter release (with some probability) from all boutons on an excited axon. Given that ACh promotes action-potential-dependent glutamate release, it is plausible that ACh may promote synchronous input to populations of neurons innervated by a common set of axons. To test whether this was the case in the 19 IT–PT dual recordings described above, a 0.5 ms window was used to identify “synchronous” events occurring in both neurons (Fig. 8*A,B*). The probability of synchronous events in baseline conditions was low (mean ± SD of 0.84 ± 1.45% of the total number of sEPSPs; *n* = 19 IT–PT pairs) but was greater than double the “chance” level of synchronous events occurring within a 0.5 ms window (0.35 ± 0.07%; *n* = 19 experimental pairs, each experiencing 36 simulated IT–PT pairings; Fig. 8*C*, Table 8; see Methods). Application of ACh failed to enhance synchronous input probabilities in IT–PT pairs (0.80 ± 1.12% of sEPSPs) and had only a small effect in simulated IT–PT pairs (0.43 ± 0.09% in the presence of ACh; Fig. 8*C*), reflecting the increase in total sEPSP occurring in ACh conditions (particularly in PT neurons). These data demonstrate that ACh does not enhance the likelihood of synchronous excitatory input in pairs of IT and PT neurons.

**Table 8.** Effects of ACh on the probability of synchronous input recorded in heterotypic and homotypic neuron pairs

Group	*n*	Mean % synchronous sEPSPs			Raw change in ACh	ACh vs baseline^c^ (*p*-value)	Effect size (*d)*
		Baseline	+ACh	Wash			
IT–IT pairs	12^a^	0.37 ± 0.25	0.83 ± 0.85	0.24 ± 0.22	+0.5 ± 1.0%	0.125	0.73
IT–IT simulated	12 (x20)^b^	0.36 ± 0.04	0.44 ± 0.09	0.27 ± 0.04	+0.1 0.1%	0.002	**1.26**
Pairs vs simulated	*t* test^c^ (*p*)	0.859	0.141	0.633	0.205		
	Effect size (*d*)	0.07	0.64	0.18	0.55		
IT–PT pairs	19^a^	0.84 ± 1.45	0.80 ± 1.12	0.31 ± 0.34	0 ± 0.8%	0.840	0.03
IT–PT simulated	19 (×36)^b^	0.35 ± 0.07	0.43 ± 0.09	0.24 ± 0.05	+0.8 ± 0.1%	0.002	**0.99**
Pairs vs simulated	*t* test^c^ (*p*)	0.149	0.175	0.341	0.534		
	Effect size (*d*)	0.48	0.46	0.30	0.20		
PT–PT pairs	22^a^	0.60 ± 0.36	2.09 ± 2.11	0.76 ± 1.02	+1.5 ± 2.1%	0.003	**0.98**
PT–PT simulated	22 (x84)^b^	0.42 ± 0.05	0.67 ± 0.05	0.35 ± 0.06	+0.3 ± 0.05%	<0.001	**4.92**
Pairs vs simulated	*t* test^c^ (*p*)	0.023	0.005	0.075	0.011		
	Effect size (*d*)	0.70	**0.95**	0.57	**0.85**		

Data are shown as means ± SD for the proportion (%) of synchronous (occurring within an 0.5 ms window) sEPSPs relative to all sEPSPs in the final 4 min each of baseline recording, ACh exposure, and wash. Large effect sizes (≥∼0.80) are shown in bold.

^a^ Percentage of synchronous sEPSPs detected in each experimental condition were averaged for each dual recording (i.e., pairs treated as “*n*”).

^b^ For each recorded pair, the percentage of “synchronous” sEPSPs detected in simulated pairings of every possible appropriate (IT–IT, IT–PT, or PT–PT) combination across both neurons were averaged (i.e., average proportion of “synchronous” events across all possible simulated pairings involving either neuron in a dual recording = *n*; see Methods).

^c^ Student's *t* test for paired samples.

To test whether ACh might promote synchronous excitatory input in homotypic pairs of neurons, additional dual recordings were made from IT–IT (*n* = 12) and PT–PT (*n* = 22) pairs (Fig. 8*C*, Table 8). In IT–IT pairs, ACh increased the probability of synchronous sEPSPs from 0.37 ± 0.25% (SD) of sEPSPs in baseline conditions to 0.83 ± 0.85% in the final 4 min of ACh exposure. This compared to a smaller increase (from 0.36 ± 0.04% to 0.44 ± 0.09%) of chance “synchronous” sEPSPs from simulated pairings (*n* = 12 dual-recording pairs each experiencing 20 simulated IT–IT pairings; Table 8) that reflects the small increase in sEPSP frequencies observed in IT neurons exposed to ACh (see Fig. 3 and Table 3). Notably, while the proportions of synchronous sEPSPs in IT–IT dual recordings and simulated IT–IT pairings were almost identical in baseline and wash conditions, in the presence of ACh the proportion of synchronous events in IT–IT dual recordings was almost double that observed for simulated pairings (Table 8), demonstrating that, unlike in IT–PT pairs, ACh modestly facilitates synchronous input in IT–IT pairs.

ACh increased the proportion of synchronous sEPSPs to a much greater extent in PT–PT dual recordings (*n* = 22) (Fig. 8*C*, Table 8). Whereas the proportion of synchronous sEPSPs in PT–PT dual recordings in baseline conditions was about 50% greater than the chance level determined from simulated pairings (0.60 ± 0.36% vs 0.42 ± 0.05%, respectively), the addition of ACh increased the fraction of synchronous sEPSPs in PT–PT dual recordings to 2.09 ± 2.11%, such that it was three times that observed in simulated PT–PT pairings (0.67 ± 0.05%). When comparing the change in the incidence of synchronous sEPSPs occurring in ACh relative to baseline, PT–PT dual recordings showed larger mean changes (+1.49 ± 2.07) than observed in IT–IT (0.46 ± 0.96; *p* = 0.057, *d* = 0.58 relative to PT–PT pairs) or IT–PT (−0.04 ± 0.83; *p* = 0.004, *d* = 0.94 relative to PT–PT pairs) pairs. These data demonstrate that ACh preferentially promotes the occurrence of synchronous sEPSPs in PT neurons.

It is worth noting that, despite testing for unitary connectivity in all dual recordings in this study, no unitary connections were identified, regardless of which projection neuron subtypes were targeted (Jiang et al., 2015). This may reflect a developmental reduction in unitary connectivity in adult animals (Jiang et al., 2015), or the occurrence of axon severing in coronal slices of prefrontal cortex, as slices were placed such that apical dendrites were level with, or descended into, the slice to preserve dendritic architecture. A consequence of this is that axons of neurons close to the surface of the slice are likely severed within a few hundred µm from the soma.

In addition to measuring the impact of ACh on synchronous inputs in PT–PT neuron pairs (above), the effects of ACh on sEPSP attributes were compared across neuron subtypes in the 12 IT–IT (24 total IT neurons) and 22 PT–PT (44 total neurons) dual recordings described above. As was found in IT–PT pairs, ACh preferentially enhanced sEPSP amplitudes (by 56 ± 9%), frequencies (by 78 ± 10%), rise-times (by 26 ± 4%), and half-widths (by 12 ± 2%) in PT neurons (*n* = 44; *p* values at or below 0.004 and effect sizes ranging from 0.76 to 1.28 when compared to the 24 IT neurons from IT–IT pairs; Fig. 8*D–H*; Table 9). Similarly, the holding currents necessary to maintain neurons at their resting *V_M_* were much larger in PT neurons (−110 ± 10 pA) than in IT neurons (−29 ± 5 pA). Thus, the results from dual IT–IT and PT–PT recordings (68 neurons in total) represent a replication of the ACh effects observed in the original 19 IT–PT dual recordings (compare Figs. 3*E–I* with 8*D–H*), providing additional confidence that cholinergic enhancement of sEPSPs occurs preferentially in PT neurons.

**Table 9.** Effects of ACh on sEPSPs in IT and PT neurons recorded in homotypic pairs

Measurement	Neuron type	Baseline	+ACh	Wash	Change in ACh	ACh vs baseline^a^ (*p*-value)	Effect size (*d*)
sEPSP amplitude (mV)	IT (24)	0.55 ± 0.04	0.61 ± 0.03	0.55 ± 0.04	+16 ± 6%	0.112	0.35
	PT (44)	0.68 ± 0.02	1.02 ± 0.05	0.74 ± 0.05	+56 ± 10%	<0.001	**1.34**
	IT–PT *t* test^b^ (*p*)	0.006	<0.001	0.003	0.001		
	Effect size (*d*)	**0.82**	**1.42**	0.70	**0.76**		
sEPSP frequency (Hz)	IT (24)	3.9 ± 0.2	4.2 ± 0.3	2.8 ± 0.2	+12 ± 10%	0.338	0.22
	PT (44)	4.2 ± 0.2	6.9 ± 0.2	3.7 ± 0.2	+78 ± 10%	<0.001	**2.10**
	IT–PT *t* test^b^ (*p*)	0.132	<0.001	<0.001	<0.001		
	Effect size (*d*)	0.39	**1.84**	**0.85**	**1.02**		
sEPSP rise-time (ms)	IT (24)	1.72 ± 0.06	1.66 ± 0.08	1.75 ± 0.09	−2 ± 4%	0.421	0.17
	PT (44)	1.64 ± 0.04	2.03 ± 0.04	1.71 ± 0.04	+26 ± 4%	<0.001	**1.35**
	IT–PT *t* test^b^ (*p*)	0.379	<0.001	0.675	<0.001		
	Effect size (*d*)	0.23	**1.12**	0.12	**1.28**		
sEPSP half-width (ms)	IT (24)	13.3 ± 0.4	13.5 ± 0.3	13.0 ± 0.4	+2 ± 3%	0.600	0.11
	PT (44)	12.6 ± 0.2	14.0 ± 0.1	13.5 ± 0.2	+12 ± 2%	<0.001	**1.24**
	IT–PT *t* test^b^ (*p*)	0.147	0.112	0.286	0.004		
	Effect size (*d*)	0.42	0.48	0.29	**0.82**		
Holding current (pA)	IT (24)	+4 ± 6	−25 ± 9	+11 ± 19	−29 ± 5 pA	<0.001	**0.77**
	PT (44)	−5 ± 3	−114 ± 10	−6 ± 9	−110 ± 10 pA	<0.001	**2.23**
	IT–PT *t* test^b^ (*p*)	0.171	<0.001	0.427	<0.001		
	Effect size (*d*)	0.41	**1.50**	0.23	**1.49**		

Data are shown as means ± SEM. Large effect sizes (≥∼0.80) are shown in bold.

^a^ Student's *t* tests for paired samples were used to compare sEPSP attributes across baseline and cholinergic conditions.

^b^ Student's *t* tests assuming unequal variances were used for comparisons of sEPSP attributes in IT and PT neurons for each experimental condition.

### Silencing PT neurons blocks cholinergic enhancement of excitatory drive

The results described above demonstrate that ACh enhances action-potential-dependent glutamate release preferentially onto PT neurons in the mouse mPFC. What might the mechanism be for this effect? A number of observations suggest that cholinergic excitation of PT neurons, as a population, could explain the selective impact of ACh on sEPSPs in those same neurons. First, the M1 receptors implicated in cholinergic enhancement of sEPSP amplitudes and frequencies also preferentially promote persistent action potential generation in PT neurons (Gulledge et al., 2009; Dembrow et al., 2010; Joshi et al., 2016; Baker et al., 2018b), and M1-dependent activation of populations of synaptically coupled neurons is expected to generate TTX-sensitive sEPSPs within that network. Second, PT axons preferentially innervate other PT neurons, rather than IT neurons, in local cortical circuits (Morishima and Kawaguchi, 2006; Brown and Hestrin, 2009; Kiritani et al., 2012), which could explain why cholinergic enhancement of excitatory drive is preferential to PT neurons. Finally, unitary PT–PT synaptic connections tend to be larger than unitary connections in IT–IT or IT–PT pairs (Morishima and Kawaguchi, 2006; Morishima et al., 2011), which would promote larger mean sEPSP amplitudes in PT neurons during cholinergic stimulation.

**Figure 9.**
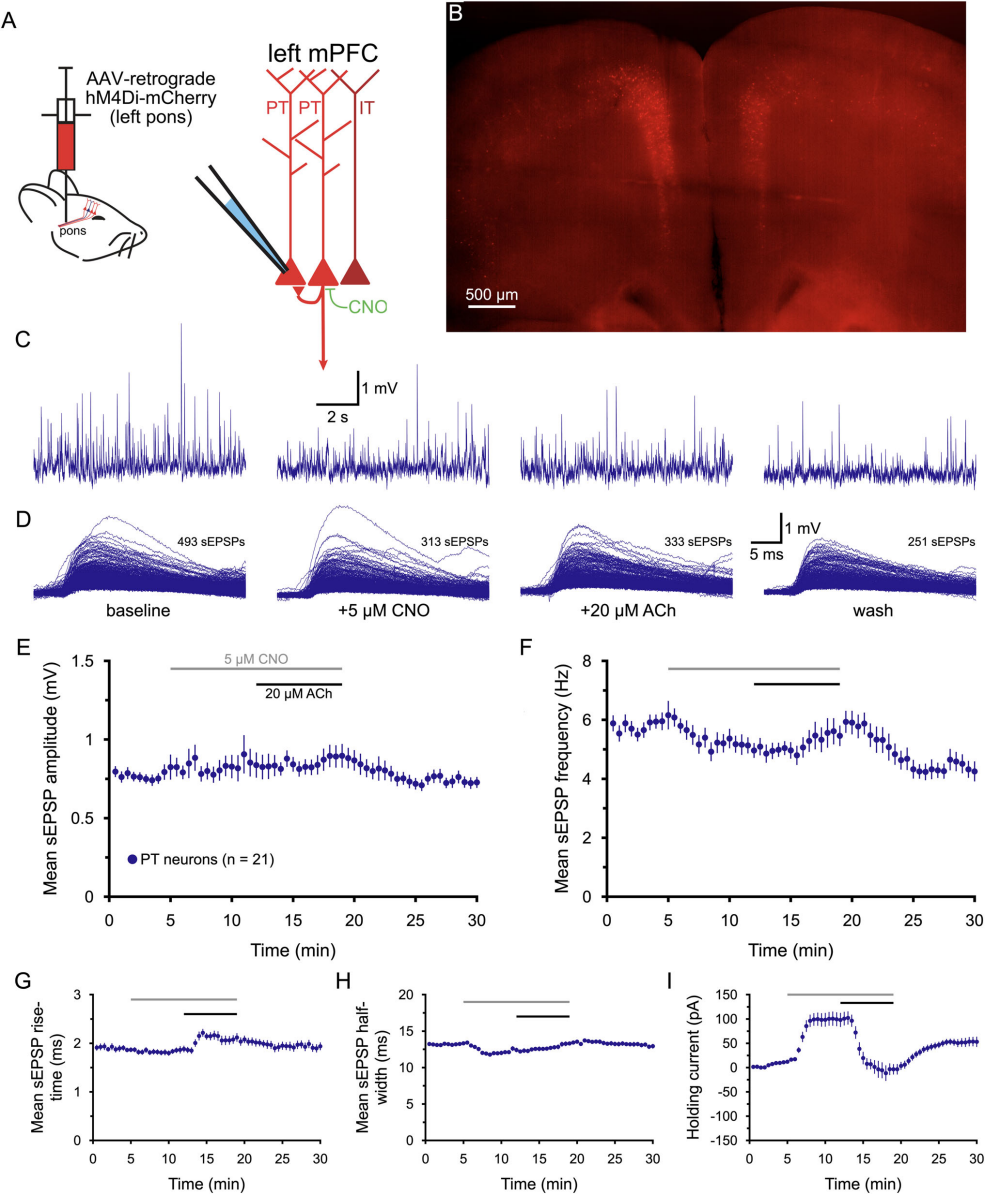
Chemogenetic inhibition of corticopontine PT neurons blocks cholinergic enhancement of excitatory drive. ***A***, Diagrams of injection of AAV-retrograde hM4Di-mCherry virus into the pons to express hM4Di receptors selectively in PT neurons in the cortex (left) and of recording setup using CNO (5 µM) to silence PT neurons (right). ***B***, Image of hM4Di-m-Cherry-expressing layer 5 PT neurons in the medial prefrontal cortex (5x objective, artificially colored) 3 weeks after virus injection into the pons. ***C***, Ten-second-long traces of membrane potential from an hM4Di-mCherry-expressing PT neuron taken at the end of the baseline recording period (far left), after 7 min of exposure to 5 µM CNO (to activate hM4Di receptors; middle left), after an additional 7 min of exposure to CNO and 20 µM ACh (with 10 µM eserine; middle right), and after 11 min of wash (far right), as indicated in ***D***. ***D***, Superimposed sEPSPs detected in the final 1-min periods of baseline recording, after CNO application, following the additional application of ACh, and after wash for the neuron shown in ***C***. ***E***,***F***, Plots of mean sEPSP amplitude (***E***) and frequency (***F***) over time for 21 PT neurons exposed to CNO and ACh (30 s means). ***G***–***I***, Plots of mean sEPSP rise-time (***G***), width at half-amplitude (***H***), and the holding current necessary to keep neurons at their resting membrane potentials (***I***) over time. Note that CNO induced a large outward current before ACh induced its own inward current, and that ACh induced an increase in sEPSP rise-times and holding currents but had no effect on sEPSP amplitudes, frequencies, or half-widths (compare with Fig. 7*C–G*).

To test whether recurrent network activity in populations of PT neurons contributes to the cholinergic increase in sEPSPs in PT neurons, a retrograde AAV virus encoding the G_i_-coupled inhibitory hM4Di receptor was injected into the pons of mice 3 weeks before experiments to allow targeted silencing of brainstem-projecting PT neurons (Fig. 9), which include a population of PT neurons projecting to the thalamus (Guo et al., 2017; Economo et al., 2018). Bath application of CNO (5 µM; an agonist for hM4Di receptors) was effective in inhibiting PT neurons, as evident from the large positive holding currents (+99 ± 14 pA; *n* = 21 PT neurons) necessary to maintain neurons at their initial resting *V_M_* (Fig. 9*I*, Table 10). CNO on its own had little impact on sEPSP amplitudes or rise-times but modestly decreased sEPSP frequencies (by 14 ± 4%; *n* = 21; *p* < 0.001, *d* = 0.71) and half-widths (by 5.8 ± 1.9%; *p* = 0.006, *d* = 0.65). In the continued presence of CNO, application of ACh activated inward currents in PT neurons (−102 ± 11 pA, returning holding currents to near zero) and increased sEPSP rise-times (by 13 ± 4%), indicating relatively normal postsynaptic actions of ACh (e.g., compare to Fig. 7*E,G*). However, CNO abolished cholinergic enhancement of sEPSP amplitudes and frequencies (Fig. 9*C–H*, Table 10), suggesting that the increase in excitatory synaptic drive stimulated by ACh require action potential generation in networks of interconnected PT neurons.

**Table 10.** Effects of ACh on sEPSPs after chemogenetic silencing of corticopontine PT neurons (*n* = 21 PT neurons)

Measurement	Baseline	+CNO	+ACh	Wash	Change in ACh	ACh vs CNO^a^ (*p*-value)	Effect size (*d*)
sEPSP amplitude (mV)	0.81 ± 0.06	0.85 ± 0.08	0.89 ± 0.07	0.73 ± 0.03	+10 ± 6%	0.512	0.14
sEPSP frequency (Hz)	6.1 ± 0.4	5.0 ± 0.2	5.5 ± 0.4	4.3 ± 0.3	+13 ± 8%	0.163	0.34
sEPSP rise-time (ms)	1.86 ± 0.05	1.87 ± 0.07	2.10 ± 0.09	1.92 ± 0.08	+13 ± 4%	0.013	0.63
sEPSP half-width (ms)	13.3 ± 0.2	12.5 ± 0.3	13.3 ± 0.3	12.9 ± 0.3	+7 ± 2%	0.002	0.55
Holding current (pA)	+11 ± 3	+99 ± 14	−3 ± 11	+53 ± 10	−102 ± 11 pA	<0.001	**1.73**

Data are shown as means ± SEM. Large effect sizes (≥∼0.80) are shown in bold.

^a^ Student's *t* test for paired samples.

**Figure 10.**
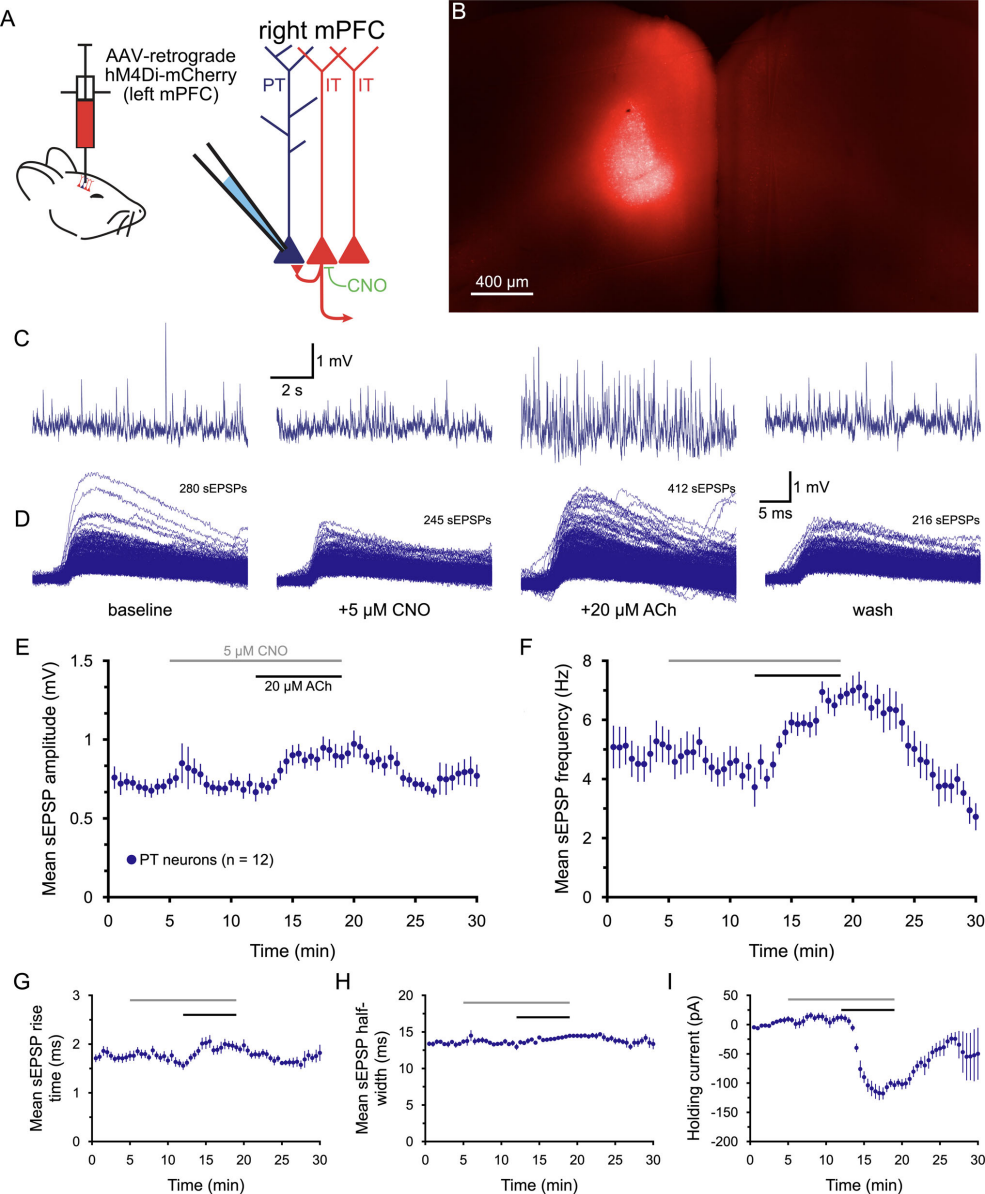
Chemogenetic inhibition of commissural IT neurons in the medial prefrontal cortex (mPFC) does not block cholinergic enhancement of excitatory drive to PT neurons, ***A***, Diagrams of injection of AAV-retrograde hM4Di-mCherry virus into the left mPFC (left) and of the recording setup using clozapine N-oxide (CNO; 5 µM) to silence IT neurons in the contralateral hemisphere (right). ***B***, Image of hM4Di-m-Cherry-expression in the mPFC (5x objective, artificially colored) 3 weeks after virus injection into the left mPFC. ***C***, Ten-second-long traces of membrane potential from a PT neuron taken at the end of the baseline recording period (far left), after 7 min of exposure to 5 µM CNO (to activate hM4Di receptors; middle left), after an additional 7 min of exposure to CNO and 20 µM ACh (with 10 µM eserine; middle right), and after 11 min of wash (far right), as indicated in ***D***. ***D***, Superimposed sEPSPs detected in the final 1-min periods of baseline recording, after CNO application, following the additional application of ACh, and after wash for the neuron shown in ***C***. ***E***,***F***, Plots of mean sEPSP amplitude (***E***) and frequency (***F***) over time for 12 PT neurons exposed to CNO and ACh (30 s means). ***G***–***I***, Plots of mean sEPSP rise-time (***G***), width at half-amplitude (***H***), and the holding current necessary to keep neurons at their resting membrane potentials (***I***) over time.

In another group of animals, the retrograde AAV hM4Di virus was injected into the left mPFC to express hM4Di in commissural (i.e., callosal projection) IT neurons in the contralateral cortex (Fig. 10*A*,*B*). Recordings of sEPSPs were then made in PT neurons in the right hemisphere. Application of 5 µM CNO had little effect on sEPSP amplitudes or holding currents, but modestly reduced sEPSP frequencies by 17 ± 6% (*n* = 12; *p* = 0.027, *d* = 0.60; Fig. 10*C–I*; Table 11). Application of ACh in the continued presence of CNO enhanced sEPSP amplitudes (by 33 ± 8%), frequencies (by 85 ± 22%), rise-times (by 26 ± 7%), and half-widths (by 10 ± 2%; Fig. 10*C–I*, Table 11). ACh also induced depolarizing postsynaptic currents (mean change in holding current was −114 ± 9 pA). The effects of ACh on sEPSPs in PT neurons after chemogenetic silencing of commissural IT neurons were of similar magnitude to those observed in PT neurons in control conditions (compare results in Table 11 with those in Table 3; *p* ≥ 0.22, *d* ≤ 0.38). These results suggests that commissural IT neurons may not contribute to cholinergic enhancement of sEPSPs in PT neurons but cannot rule out involvement of other IT neuron subtypes that do not target the contralateral prefrontal cortex.

**Table 11.** Effects of ACh on sEPSPs after chemogenetic silencing of commissural IT neurons (*n* = 12 PT neurons)

Measurement	Baseline	+CNO	+ACh	Wash	Change in ACh	ACh vs CNO^a^ (*p*-value)	Effect size (*d*)
sEPSP amplitude (mV)	0.70 ± 0.05	0.68 ± 0.06	0.82 ± 0.03	0.76 ± 0.07	+33 ± 8%	0.058	**0.85**
sEPSP frequency (Hz)	5.0 ± 0.7	3.9 ± 0.4	6.5 ± 0.4	3.0 ± 0.4	+85 ± 22%	0.002	**1.94**
sEPSP rise-time (ms)	1.74 ± 0.09	1.58 ± 0.08	1.95 ± 0.09	1.79 + 0.15	+26 ± 7%	0.005	**1.23**
sEPSP half-width (ms)	13.5 ± 0.3	13.2 ± 0.3	14.4 ± 0.2	13.4 ± 0.6	+10 ± 2	0.001	**1.44**
Holding current (pA)	+9 ± 5	+12 ± 6	−102 ± 7	−51 ± 42	−114 ± 9 pA	<0.001	**4.86**

Data are shown as means ± SEM. Large effect sizes (≥∼0.80) are shown in bold.

^a^ Student's *t* test for paired samples.

**Figure 11.**
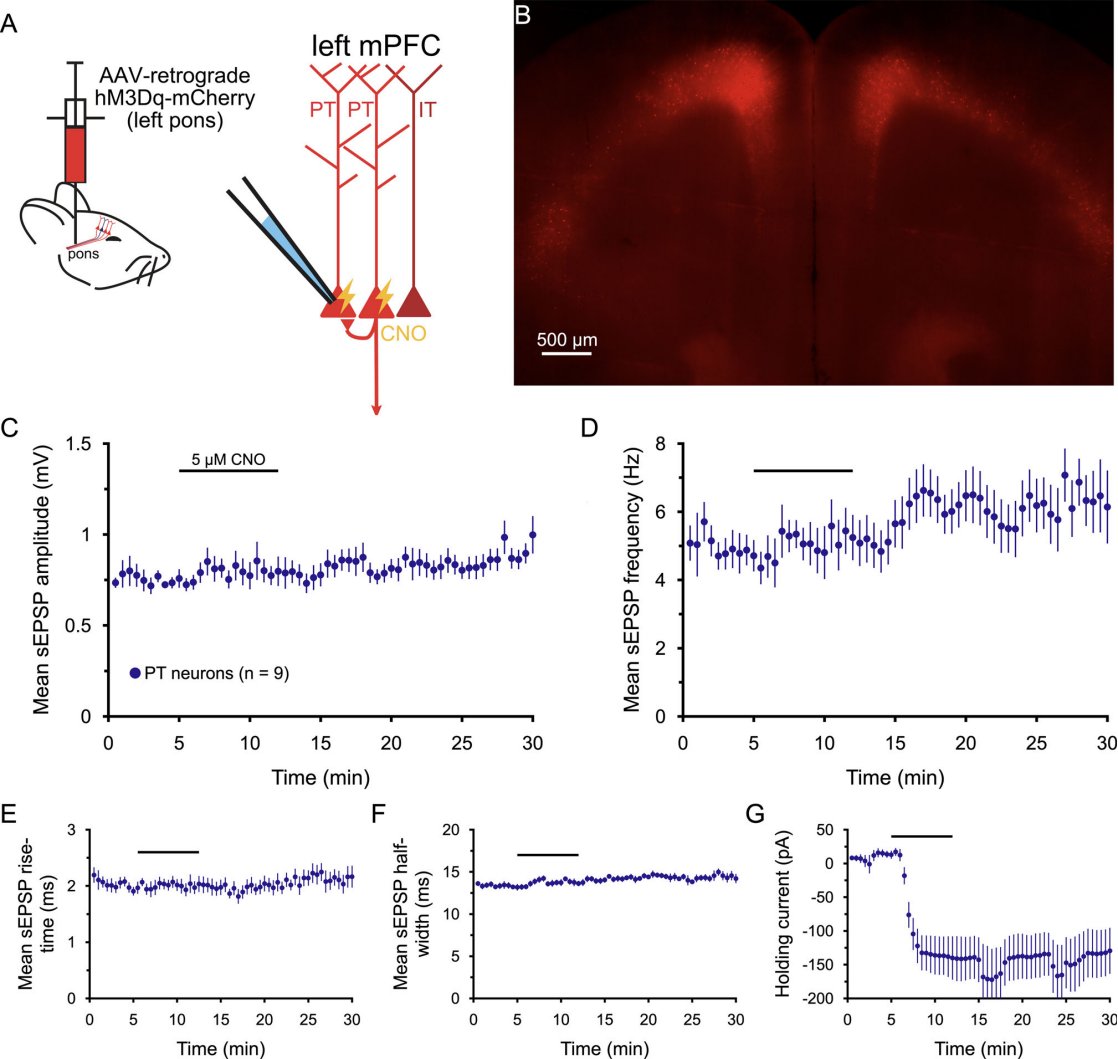
The effects of ACh on sEPSPs are not mimicked by activation of hM3Dq receptors in corticopontine PT neurons. ***A***, Diagrams of injection of AAV-retrograde hM3Dq-mCherry virus into the pons to express hM3Dq receptors selectively in PT neurons in the cortex (left) and of the recording setup using clozapine N-oxide (CNO; 5 µM) to excite PT neurons (right). ***B***, Image of hM3Dq-m-Cherry-expressing layer 5 PT neurons in the prefrontal cortex (5x objective, artificially colored) 3 weeks after virus injection into the pons. ***C***, Plot of mean sEPSP amplitude (average of 30 s means ± SEM) over time for 9 PT neurons expressing hM3Dq-mCherry. CNO was bath-applied for 7 min, as indicated. ***D***–***G***, Similar to ***C***, with plots of mean sEPSP frequency (***D***), rise-time (***E***), and half-width (***F***) over time. ***G***, Plot of the mean holding current necessary to maintain neurons at their initial resting membrane potentials. Note that CNO induced large and irreversible inward currents (***G***) but had no effect on sEPSP properties, including sEPSP rise-times (***E***).

The chemogenetic experiments described above demonstrate that network activity in PT, but not commissural IT, neurons is necessary for cholinergic enhancement of sEPSPs in PT neurons. To test whether selective excitation of PT neuron populations might be sufficient to mimic the effects of ACh on sEPSPs, a retrograde AAV encoding the G_q_-coupled DREADD hM3Dq was injected into the left pons (Fig. 11*A*,*B*). Three weeks later, recordings of sEPSPs were made in hM3Dq^+^ PT neurons in the ipsilateral mPFC (*n* = 9). Although bath application of CNO (5 µM) led to extremely large and irreversible (within 20 min) holding currents in PT neurons (mean of −152  29 pA; Fig. 11*G*, Table 12), CNO had little if any effect on sEPSPs (Fig. 11*C–F*, Table 12). The lack of effect of CNO on sEPSP rise-times, an otherwise postsynaptic effect of ACh (see Fig. 7), suggests that CNO-induced activation of hM3Dq in PT neurons does not fully mimic M1 receptor stimulation. This may reflect differences in the subcellular localizations of DREADD and M1 receptors, or over-activation of G_q_-coupled cascades in hM3Dq-expressing neurons. For instance, broad AAV-mediated expression of hM3Dq in PT neurons, including within axons (Mitew et al., 2018; Liang et al., 2020) that are normally devoid of M1 receptors (Oda et al., 2018), could lead to depolarization block (Yi et al., 2015) and impaired synaptic transmission at PT terminals. These considerations complicate interpretation of this negative result following hM3Dq activation in PT neurons. Alternative approaches will be necessary to determine whether selective activation of PT neurons is sufficient to mimic the effect of ACh on sEPSPs.

**Table 12.** Effects of chemogenetic activation of corticopontine PT neurons on sEPSPs (*n* = 9 PT neurons)

Measurement	Baseline	+CNO	Wash	Change in CNO	CNO vs baseline^a^ (*p*-value)	Effect size (*d*)
sEPSP amplitude (mV)	0.75 ± 0.04	0.79 ± 0.07	0.94 ± 0.7	+5 ± 6%	0.422	0.24
sEPSP frequency (Hz)	4.8 ± 0.5	5.3 ± 0.7	6.3 ± 1.0	+17 ± 14%	0.656	0.30
sEPSP rise-time (ms)	1.94 ± 0.08	2.01 ± 0.13	2.16 ± 0.19	+3 ± 4%	0.397	0.20
sEPSP half-width (ms)	13.2 ± 0.3	13.7 ± 0.4	14.4 ± 05	+4 ± 2%	0.110	0.45
Holding current (pA)	+13 ± 4	−139 ± 46	−131 ± 44	−152 ± 29 pA	<0.001	**2.28**

Data are shown as means ± SEM. Large effect sizes (≥∼0.80) are shown in bold.

^a^ Student's *t* test for paired samples.

## Discussion

### ACh enhances excitatory drive preferentially in PT neurons

Given the wealth of data demonstrating cholinergic suppression of glutamate release at excitatory cortical synapses (see Introduction), ACh was expected to reduce excitatory synaptic drive in both IT and PT neurons. The results described above demonstrate that this is not the case. Instead, in two independent data sets, ACh promoted excitatory drive preferentially in PT neurons (see Figs. 3, 8), an effect that was robust across sEPSP detection thresholds and the kinetics of the template used to detect sEPSPs (see Fig. 3*E*,*F*). The sensitivity of cholinergic enhancement of sEPSP amplitudes and frequencies to TTX (see Fig. 7) demonstrates that ACh acts at the network level to enhance excitatory drive in PT neurons. The reduction of mEPSP frequencies, but not amplitudes, by ACh in the presence of TTX suggests that glutamate release is suppressed by ACh at many cortical terminals, likely via presynaptic M4 receptors, as observed in young layer 2/3 neurons (Cho et al., 2019) and layer 6 IT neurons (Yang et al., 2020) in rat sensory cortex (Kimura and Baughman 1997; Eggermann and Feldmeyer, 2009; Yang et al., 2020; see also Shirey et al., 2008; Dasari and Gulledge, 2011).

Cholinergic facilitation of excitatory drive in PT neurons is noteworthy because ACh also preferentially enhances PT neuron excitability, as reflected in the larger holding currents necessary to keep PT neurons at their initial resting *V_M_* (e.g., Figs. 3*H*, 8*H*), a result consistent with prior reports of selective cholinergic excitation of PT neurons (Dembrow et al., 2010; Joshi et al., 2016; Baker et al., 2018b). The present results are also consistent with those of Shirey et al. (2009), who found that M1 receptor activation enhanced the amplitude and frequency of spontaneous, but not miniature, EPSPs in layer 5 neurons in the mPFC of young (< 4week old) rodents. The present study goes further in demonstrating that the cholinergic increase in excitatory drive is preferential to PT neurons and requires recurrent excitation within networks of PT neurons (see Figs. 3, 8, 9). Thus, ACh release in the cortex engages two parallel mechanisms that promote corticofugal output to the brainstem: preferential increase of PT neuron excitability, and robust recurrent excitatory drive within PT networks.

Cholinergic enhancement of synaptic drive in PT neurons is attributed to M1-type muscarinic receptor activation, as it was blocked by both atropine (a broad spectrum mAChR antagonist; see Fig. 4) and pirenzepine (an M1-selective antagonist; see Fig. 5, Shirey et al., 2009). It is notable that, after selective blockade of M1 receptors, ACh moderately *decreased* sEPSP frequency in PT neurons, a result that aligns with the well-established M4-dependent presynaptic inhibition of glutamate release at neocortical (Kimura and Baughman, 1997; Eggermann and Feldmeyer, 2009; Yang et al., 2020) and hippocampal (Shirey et al., 2008; Dasari and Gulledge, 2011) synapses. Cholinergic enhancement of PT–PT recurrent activity occurring in parallel with generalized suppression of glutamatergic transmission may further focus corticofugal output within populations of synaptically-coupled PT neurons.

Activation of mAChRs can also increase the amplitude and frequency of action-potential-dependent inhibitory postsynaptic currents (IPSCs) in cortical pyramidal neurons (Kawaguchi, 1997; Kondo and Kawaguchi, 2001), potentially via direct excitation of somatostatin-expressing GABAergic interneurons (Kawaguchi, 1997; Fanselow et al., 2008; Xu et al., 2013; Chen et al., 2015; Obermayer et al., 2018). On the other hand, ACh suppresses GABA release at perisomatic inhibitory synapses made by fast-spiking interneurons (Kruglikov and Rudy, 2008) (Aramakis et al., 1997; Patil and Hasselmo, 1999; Salgado et al., 2007; Nunez et al., 2012). These results suggest ACh may enhance inhibition to tuft dendrites while simultaneously disinhibiting perisomatic regions of pyramidal neurons. In the present study, cholinergic enhancement of sEPSPs was similar in control conditions with intact GABAergic networks and in conditions in which GABAergic synaptic transmission was blocked (see Fig. 6), demonstrating that cholinergic modulation of inhibitory circuits is not required to enhance the excitatory synaptic drive of PT neurons in the adult prefrontal cortex. As most of the prior studies described above utilized immature (pre-weening) rodents (but see Chen et al., 2015), it is possible that there are developmental changes in the cholinergic sensitivity of inhibitory cortical circuits that remain to be defined. Alternatively, because PT neurons typically establish perisomatic connections with other PT neurons in the local network (Morishima et al., 2011), and inputs from ACh-sensitive somatostatin neurons typically occur in distal apical dendrites (Silberberg and Markram, 2007), there may be limited interaction among the two inputs. Regardless, enhanced inhibition of the apical tuft is expected to further shift the weight of excitatory drive toward more proximal PT–PT recurrent connections.

Although pyramidal neurons in the mPFC lack significant postsynaptic nicotinic responses to ACh (Hedrick and Waters, 2015), presynaptic nAChRs may modulate glutamate (Lambe et al., 2003; Couey et al., 2007; Aracri et al., 2013) and GABA (Couey et al., 2007; Aracri et al., 2010) release in the mPFC. In addition, ACh may excite a subpopulation of cortical GABAergic interneurons that express postsynaptic nAChRs (Poorthuis et al., 2014). The present study tested the impact of ACh, rather than specific cholinergic agonists, to capture the net effect of ACh on network activity in IT and PT neurons, regardless of which receptor subtypes might be involved (i.e., mAChRs or nAChRs). However, the ability of atropine to block all cholinergic effects on sEPSPs (Fig. 4) suggests that nAChRs may not regulate local network excitatory drive to adult IT and PT neurons (Aramakis and Metherate, 1998).

### ACh promotes recurrent activity in networks of PT neurons

Dual recordings allowed for the detection and measurement of synchronous sEPSPs under different experimental conditions. In baseline conditions the rate of synchronous sEPSPs was very low, either equivalent to random chance (IT–IT pairs) or somewhat above random chance (in IT–PT and PT–PT pairs). ACh preferentially increased the rate of synchronous sEPSPs in PT–PT pairs (see Fig. 8; Table 8). Given that TTX reduced sEPSP frequencies in PT neurons by 30% (see Fig. 7), it can be estimated that mEPSPs account for the remaining 70% of spontaneous events. The abundance of mEPSPs, which by nature are not purposefully synchronized across release sites, and which are not enhanced by ACh, likely occludes the full impact of cholinergic synchronization of action-potential-evoked sEPSPs in PT neurons. Therefore, it can be estimated that cholinergic stimulation synchronizes upwards of ∼7% of action-potential-dependent sEPSPs in individual PT–PT pairs. The additional excitatory drive to PT neurons in the presence of ACh is attributable to recurrent PT networks, as it was eliminated by chemogenetic silencing of corticopontine PT neurons but not commissural IT neurons (see Figs. 9, 10). Although chemogenetic excitation of PT neurons failed to mimic cholinergic enhancement of sEPSPs (Fig. 11), it also failed to reproduce other aspects of cholinergic signaling (e.g., the increase in sEPSP rise-times), making it difficult to interpret this negative finding. Alternative approaches to selectively activate PT networks will be needed to confirm whether increased PT excitability is sufficient to enhance sEPSP amplitudes and frequencies.

Despite a lack of in vivo data regarding synchronized excitatory input to PT neurons, it is notable that populations of motor cortex PT neurons that share projection targets exhibit concurrent activity during motor behavior (Olivares-Moreno et al., 2019; Nelson et al., 2021), suggesting that they also share excitatory drive. Given that PT neurons selectively form strong unitary connections with other PT neurons (Morishima and Kawaguchi, 2006; Morishima et al., 2011) and that cortical ACh release is associated with the initiation of motor activity (Day et al., 1991; Parikh et al., 2007; Jing et al., 2020; Lohani et al., 2022), it is plausible that ACh acts to guide behavior by promoting coordinated activity in ensembles of synaptically coupled PT neurons.

### Conclusions and considerations

The results of this study demonstrate that ACh, acting on M1-type mAChRs, promotes corticofugal output by enhancing recurrent excitation in networks of PT neurons. This study focused on comparing the net impact of ACh on local network synaptic drive to IT an PT neurons. However, many excitatory synapses arise from long-distance corticocortical and thalamocortical afferents that are not expected to contribute to action-potential-dependent EPSPs in the ex vivo conditions utilized here. Therefore, to fully elucidate how ACh regulates information flow in the neocortex it will be important to test the impact of ACh on evoked transmitter release from a range of cortical afferents. This has been done to a limited extent in the hippocampus, where differential presynaptic sensitivity to ACh is thought to shift the weight of synaptic drive from Shaffer collateral (CA3-to-CA1) inputs toward more distal excitatory inputs from the entorhinal cortex (Hasselmo and Schnell, 1994; Thorn et al., 2017). While there are data demonstrating afferent-specific cholinergic modulation in the neocortex (e.g., prefenential gating of thalamocortical afferents; Hasselmo and Cekic, 1996; Gil et al., 1997; Hsieh et al., 2000; Lambe et al., 2003), additional studies will be necessary to elucidate, at a fine scale, the full range of excitatory cortical afferents regulated by ACh and their relative contribution to IT and PT neuron excitatory drive.
